# Hierarchically Porous Multiphase Si‐Based Ceramics with Synergistic Electromagnetic Wave Absorption Mechanisms

**DOI:** 10.1002/advs.202510445

**Published:** 2025-08-14

**Authors:** Jiaojiao Jiang, Xiaomei Deng, Sihan Li, Xiaojun Zeng, Chunxiao Wu, Chao Yang

**Affiliations:** ^1^ State Key Laboratory of Biopharmaceutical Preparation and Delivery Institute of Process Engineering Chinese Academy of Sciences Beijing 100190 China; ^2^ School of Chemical Engineering University of Chinese Academy of Sciences Beijing 100049 China; ^3^ School of Materials Science and Engineering Jingdezhen Ceramic University Jingdezhen 333403 China

**Keywords:** electromagnetic wave absorption, hierarchical porosity, impedance matching, multiphase composites, one‐step etching activation, Si‐based ceramics

## Abstract

The development of high‐performance electromagnetic wave absorbers is critical for mitigating electromagnetic pollution in modern electronic and communication systems. Here, a scalable strategy is developed to fabricate hierarchically porous, multiphase Si‐based ceramics (Si_x_‐O_y_‐C_z_) via one‐step activation of carbon‐rich polycarbosilane precursors. The resulting material integrates β‐SiC crystals, amorphous SiOC, and conductive carbon within a tunable porous architecture. This combination creates abundant heterogeneous interfaces, defect structures, and enhanced impedance matching. The optimized sample achieves a minimum reflection loss of −70.44 dB at just 1.79 mm thickness and a broad 4.32 GHz bandwidth at a matching thickness of 1.86 mm. Structural, dielectric, and radar simulation analyses reveal that interfacial polarization, dipolar polarization, conduction loss, and pore‐induced scattering work synergistically to dissipate electromagnetic energy. This work offers a simple, cost‐effective approach to engineer next‐generation ceramic EMW absorbers.

## Graphical abstract

A novel multiphase Si‐based ceramic material with hierarchical porosity is devised via one‐step etching activation of carbon‐rich polycarbosilane ceramics. Integrating free carbon, SiC crystals, amorphous SiOC, and engineered porosity, the material surprisingly achieves exceptional electromagnetic absorption performance, offering new insights into the synergistic design of multiphase ceramics and porous architectures.

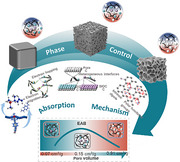

## 1 Introduction

The rapid growth of wireless communication technologies and electronic devices has intensified electromagnetic interference (EMI) and pollution, significantly affecting electronic device reliability and posing risks to human health and environmental safety.^[^ ^1^, ^2^, ^3^, ^4^ ^]^ Consequently, there is an urgent need for advanced electromagnetic wave (EMW) absorbers featuring lightweight design, thin thickness, highly efficient, and broadband absorption capabilities.^[^ ^5^, ^6^, ^7^, ^8^, ^9^ ^]^ Among potential materials, Si‐based ceramics have attracted substantial attention due to their exceptional mechanical robustness,^[^ ^10^ ^]^ thermal stability,^[^ ^11^ ^]^ and tunable dielectric properties,^[^ ^12^ ^]^ positioning them as promising candidates for next‐generation EMW absorbers.^[^ ^13^, ^14^, ^15^ ^]^

However, developing high‐performance EMW absorbers using single‐phase Si‐based ceramics remains challenging due to inherent limitations such as poor impedance matching, low intrinsic conductivity, and insufficient polarization capability. These limitations typically result in a narrow absorption bandwidth and weak attenuation properties. For instance, traditional polymer‐derived SiC ceramics exhibit a reflection loss of merely −9.9 dB at a thickness of 2.75 mm,^[^ ^16^ ^]^ underscoring the need for enhanced design strategies. To overcome these drawbacks and realize more efficient EMW absorption, extensive efforts have been devoted to incorporating additional functional phases into Si‐based ceramics. Common approaches include integrating conductive carbon phases (e.g., carbon fibers,^[^ ^17^ ^]^ carbon nanotubes,^[^ ^18^ ^]^ multiwalled carbon nanotubes,^[^ ^19^ ^]^ and graphene^[^ ^20^, ^21^ ^]^), introducing magnetic particles (including Fe,^[^ ^22^, ^23^, ^24^ ^]^ Co,^[^ ^25^, ^26^, ^27^ ^]^ Ni,^[^ ^28^, ^29^ ^]^ and their oxides^[^ ^30^, ^31^, ^32^ ^]^), and employing structural design strategies like porous architecture,^[^ ^33^, ^34^ ^]^ nanowires,^[^ ^35^, ^36^ ^]^ hollow structure,^[^ ^37^, ^38^ ^]^ aerogel^[^ ^39^, ^40^, ^41^ ^]^ and core‐shell configurations.^[^ ^42^, ^43^ ^]^ For example, Du et al.^[^ ^44^ ^]^ reported C‐doped SiC nanocomposites synthesized via a solvothermal method using glucose as carbon source, achieving a surprising reflection loss of −76.6 dB at 16.0 GHz with a thickness of only 1.66 mm. These integrating phase design approaches combine complementary electromagnetic loss mechanisms, significantly enhancing microwave attenuation performance and highlighting promising opportunities for next‐generation EMW absorbers.

Among these integrating phase design strategies, constructing porous structure within the base material has attracted considerable attention due to its straightforward fabrication, large specific surface area, controllable pore sizes, and low density.^[^ ^45^, ^46^ ^]^ Porous structure facilitates deeper penetration of electromagnetic waves into the absorber, promoting multiple reflections and scattering losses, thus significantly improving impedance matching and enhancing EMW attenuation. Recently, Bai^[^ ^47^ ^]^ et al. developed porous SiCN_nw_/C/Si_3_N_4_ ceramics through simple vacuum impregnation and heat treatment, optimizing the impedance matching and realizing efficient EMW absorption. Similarly, Zeng^[^ ^46^ ^]^ et al. prepared 3D porous carbon aerogels from pine wood via a hydrothermal and subsequent heat treatment process, obtaining an excellent EMW absorption performance with reflection loss reaching −61.6 and −58.2 dB at thicknesses of 3.7 and 1.2 mm, respectively. However, strategies focusing solely on either porous structures or multiphase compositions often fail to achieve optimal performance. Most existing methods involve complex procedures or precursors, limiting scalability and precise control over structure‐composition synergy. Moreover, few studies address the simultaneous optimization of impedance matching and dielectric loss via co‐construction of multiphase interfaces and hierarchical porosity. Therefore, developing a simple and scalable method to integrate both features is essential for advancing high‐performance EMW absorbers.

Herein, we present a novel multiphase ceramic absorber (Si_x_‐O_y_‐C_z_) inherently featuring hierarchical porosity, specifically designed for exceptional EMW absorption. The material is synthesized via a facile, scalable, and cost‐effective approach involving grafting carbon‐rich groups onto polycarbosilane precursors followed by controlled ceramization. This process simultaneously induces the formation of free carbon, crystalline SiC phases, and amorphous Si─O─C structures. Subsequently, hierarchical porosity is introduced through a simple one‐step etching activation under moderate conditions, providing abundant interfacial construction and multiple scattering sites without requiring complicated template removal or costly procedures, while achieving precise control over the composition and distribution of the multiple phases. Such simplicity and controllability not only lower the barrier for material fabrication but also make the method highly adaptable to industrial‐scale applications and real‐world deployment.

In this work, we systematically investigate the electromagnetic loss mechanisms associated with each phase and clarify how their synergistic integrations enable the Si_x_‐O_y_‐C_z_ material to achieve superior absorption efficiency across a broad frequency range. Compared to conventional phase doping or mixing strategies, the simplicity and scalability of this fabrication approach further enhance its practical applicability for EMI mitigation. Our work not only advances the fundamental understanding of multiphase ceramic absorber design but also establishes the Si_x_‐O_y_‐C_z_ material as a promising platform for next‐generation EMW absorbers with superior efficiency and practical feasibility. Taken together, this study not only expands the design toolkit for Si‐based ceramic absorbers but also bridges the gap between academic materials design and scalable technological application.

## 2 Results and Discussion

**Figure 1:**
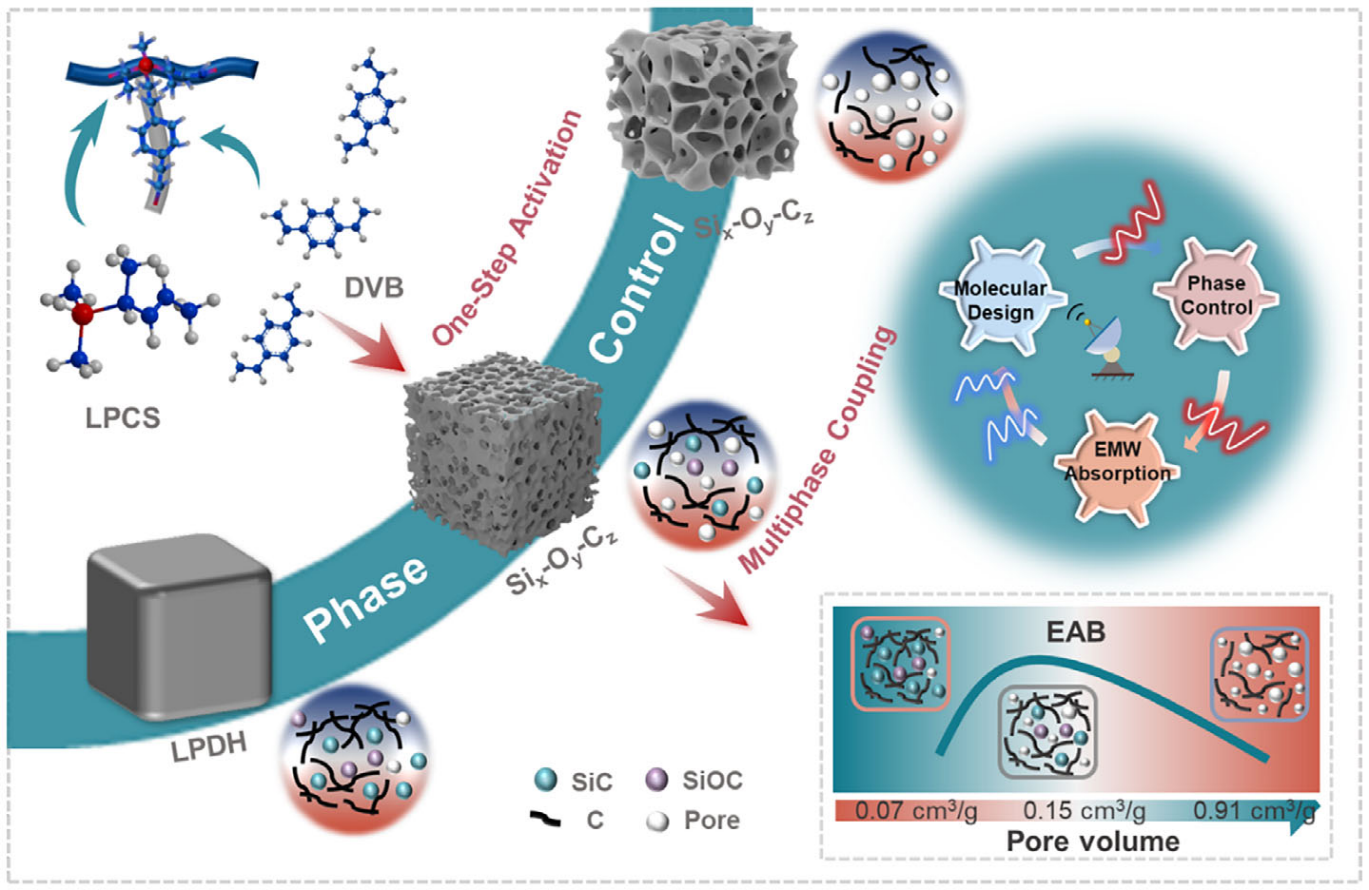
Schematic illustration of the synthesis of Si_x_‐O_y_‐C_z_ ceramics. Synergistic control of phase composition and hierarchical porosity enhances dielectric loss and impedance matching. *Note: All samples are in powder form; the macroscopic shapes shown are for illustrative purposes only*.

As illustrated in **Figure** **1**, the synthesis process begins with hydrosilylation modification of LPCS with DVB, followed by pyrolysis and alkali activation. The phase separation during ceramization yields crystalline SiC, amorphous SiOC, and disordered/graphitic carbon. Simultaneously, chemical etching induces hierarchical porosity and point defects, which promote interfacial and dipolar polarization. These microstructural features synergistically enhance dielectric loss and impedance matching, as outlined in the schematic.

**Figure 2:**
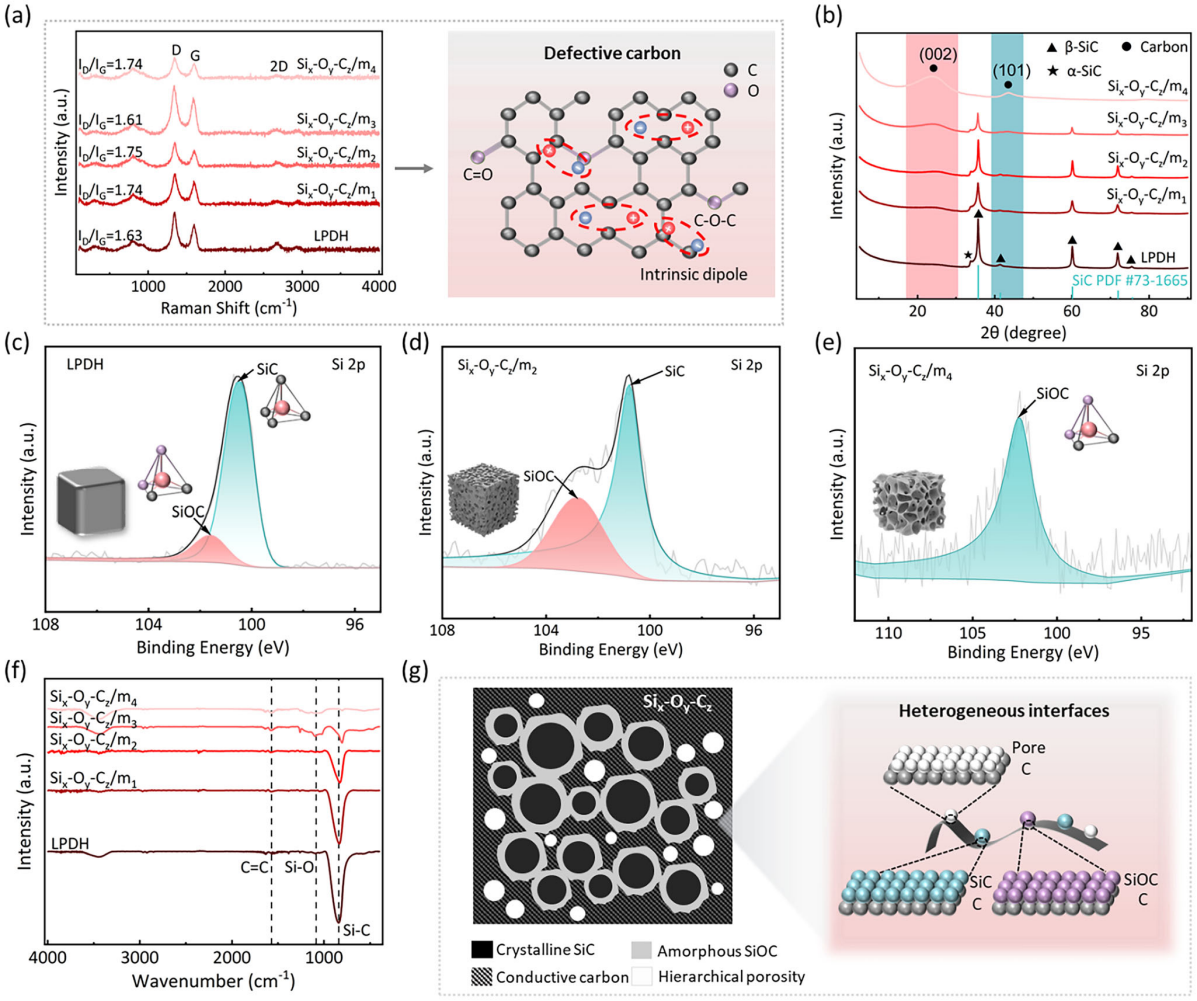
a) Raman plots and schematic illusion of defective carbon. b) XRD patterns. c–e) Si 2p XPS spectra of LPDH, Si_x_‐O_y_‐C_z_/m_2_, and Si_x_‐O_y_‐C_z_/m_4_. f) FTIR spectra of LPDH, Si_x_‐O_y_‐C_z_/m_1_, Si_x_‐O_y_‐C_z_/m_2_, Si_x_‐O_y_‐C_z_/m_3_, Si_x_‐O_y_‐C_z_/m_4_. g) Multiphase composition and schematic illusion of multiple interfaces enlargement.

**Figure** **2** illustrates the construction and structural properties of the multiphase Si_x_‐O_y_‐C_z_. The molecular architecture of the precursors was analyzed using NMR spectroscopy (Figure S1, Supporting Information). For the original LPCS, the dominant signal peak at δ = 3 ppm and minor peaks at 20–40 ppm and 130–140 ppm corresponds to CH_3_‐R, R_2_‐CH_2_/R_3_CH, and ─CH═CH─ groups, respectively. The modified polymer spectrum exhibits broad peaks between 30 and 60 ppm (R_2_‐CH_2_ and R_3_CH) and distinct resonances at 128 and 145 ppm (vinyl and phenyl groups), confirming successful grafting of DVB on LPCS (denoted as LPD). Additional structural changes, such as the reduction of ─Si─H bonds due to hydrosilylation and dehydrogenation reactions, are confirmed through FTIR (Figure S2, Supporting Information). To further tailor the material's composition and properties, the LPD was thermally cross‐linked and pyrolyzed at 1600 °C (denoted as LPDH), which served as the precursor for one‐step etching to generate hierarchical porosity. The resulting multiphase materials, denoted as Si_x_‐O_y_‐C_z_/m, where m represents the alkali‐to‐LPDH ratio (0.5, 1, 2, and 4 for Si_x_‐O_y_‐C_z_/m_1_, Si_x_‐O_y_‐C_z_/m_2_, Si_x_‐O_y_‐C_z_/m_3_, and Si_x_‐O_y_‐C_z_/m_4_, respectively), exhibit systematic variations in pore density and multiphase structure. The systematic variation in pore density and defect distribution lays the foundation for investigation of the relationship between multiphase structure and electromagnetic properties.

The structural evolution of Si_x_‐O_y_‐C_z_ is revealed through Raman, XRD, FTIR, and XPS. As shown in Figure 2a, two characteristic peaks at ≈1340 and 1590 cm^−1^ correspond to the D and G bands of carbon, respectively. The G band reflects the degree of graphitization, while the D band indicates the presence of disordered or defective carbon structures. The I_D_/I_G_ ratio initially increases due to the generation of defective carbon during the alkali activation process, and subsequently decreases as free carbon is progressively consumed. The elevated I_D_/I_G_ values in Si_x_‐O_y_‐C_z_/m₁ and Si_x_‐O_y_‐C_z_/m_2_ confirm the formation of abundant structural defects, which can serve as dipolar polarization centers to enhance electromagnetic energy dissipation. XRD spectra (Figure 2b) indicate a gradual reduction of β‐SiC peaks with increasing porosity, accompanied by the emergence of graphite peaks at 24° and 44° (graphite (002) and (101) planes). These shifts suggest partial transformation of the SiC into carbon and Si─O bonds, facilitated by strong alkali activation. The coexistence of residual crystalline β‐SiC, amorphous SiOC, free carbon, and hierarchical porous structures gives rise to extensive heterogeneous interfaces, which enhance interfacial polarization and multiple scattering.

The elemental composition and the overall electronic structural evolution of Si_x_‐O_y_‐C_z_ were investigated through XPS spectra (Figure 2c–e; Figure S3, Supporting Information). As pore density increases, Si─C bonds diminish while Si─O─C bonds dominate in Si_x_‐O_y_‐C_z_/m_4_, suggesting the transformation of SiC into carbon and Si─O bonds during the etching process. Figure 2f illustrates the molecular structural evolution of Si_x_‐O_y_‐C_z_, as analyzed via FTIR. After etching, most characteristic peaks of the original LPDH disappeared, leaving prominent absorption peaks at 840 and 3450 cm^−1^, corresponding to Si─C and O─H bonds, respectively. As pore density increases, the Si─C peak weakens, shifts to a lower wavenumber, and disappears completely in Si_x_‐O_y_‐C_z_/m_4_, consistent with the XRD results showing the breakdown of the SiC phase. Concurrently, new peaks associated with C═C and Si─O bonds emerge in Si_x_‐O_y_‐C_z_/m_3_ and Si_x_‐O_y_‐C_z_/m_4_, consistent with the structural transition observed in XPS and XRD. To better clarify the compositional logic of the Si_x_‐O_y_‐C_z_ system, schematics of its chemical bonding structure and corresponding multiphase composition are presented in Figure S4 (Supporting Information) and Figure 2g, where crystalline SiC, amorphous SiOC, and free carbon phases are interconnected. These transformations elucidate the detailed pathway of multiphase structure evolution. These changes highlight the dynamic evolution of the multiphase structure under simple one‐step etching activation.

The above analysis indicates that pore formation is consistently accompanied by the breakdown of the SiC phase, along with the emergence of graphite and ─Si─O─ bonds. This transformation is likely initiated by the alkali‐induced hydrolysis of Si─O─Si and Si─O─C bonds within the original ceramic matrix, yielding soluble silicates and in situ water at elevated temperatures. The generated water subsequently facilitates the hydrothermal decomposition of SiC – particularly at surface defects or within amorphous SiC regions – leading to the formation of silicon‐oxygen compounds, carbon, and hydrogen gas, as expressed below:

(1) $$\mathrm{SiC}+2H_{2}O\;\to \;\mathrm{Si}O_{2}+C+2H_{2}↑$$

This reaction pathway is thermodynamically favorable under alkaline conditions,^[^ ^48^, ^49^ ^]^ and is consistent with the observations by Yang et al., who reported graphite formation in NaOH‐treated SiOC at 400–700 °C.^[^ ^50^ ^]^ Furthermore, other studies have demonstrated that under strong alkaline and thermal environments, residual or disordered carbon can undergo structural reorganization and partial graphitization.^[^ ^51^, ^52^, ^53^ ^]^ This mechanism underlines the crucial role of alkali activation not only in pore formation but also in constructing multiphase microstructures with enhanced EMW absorption capability.

**Figure 3:**
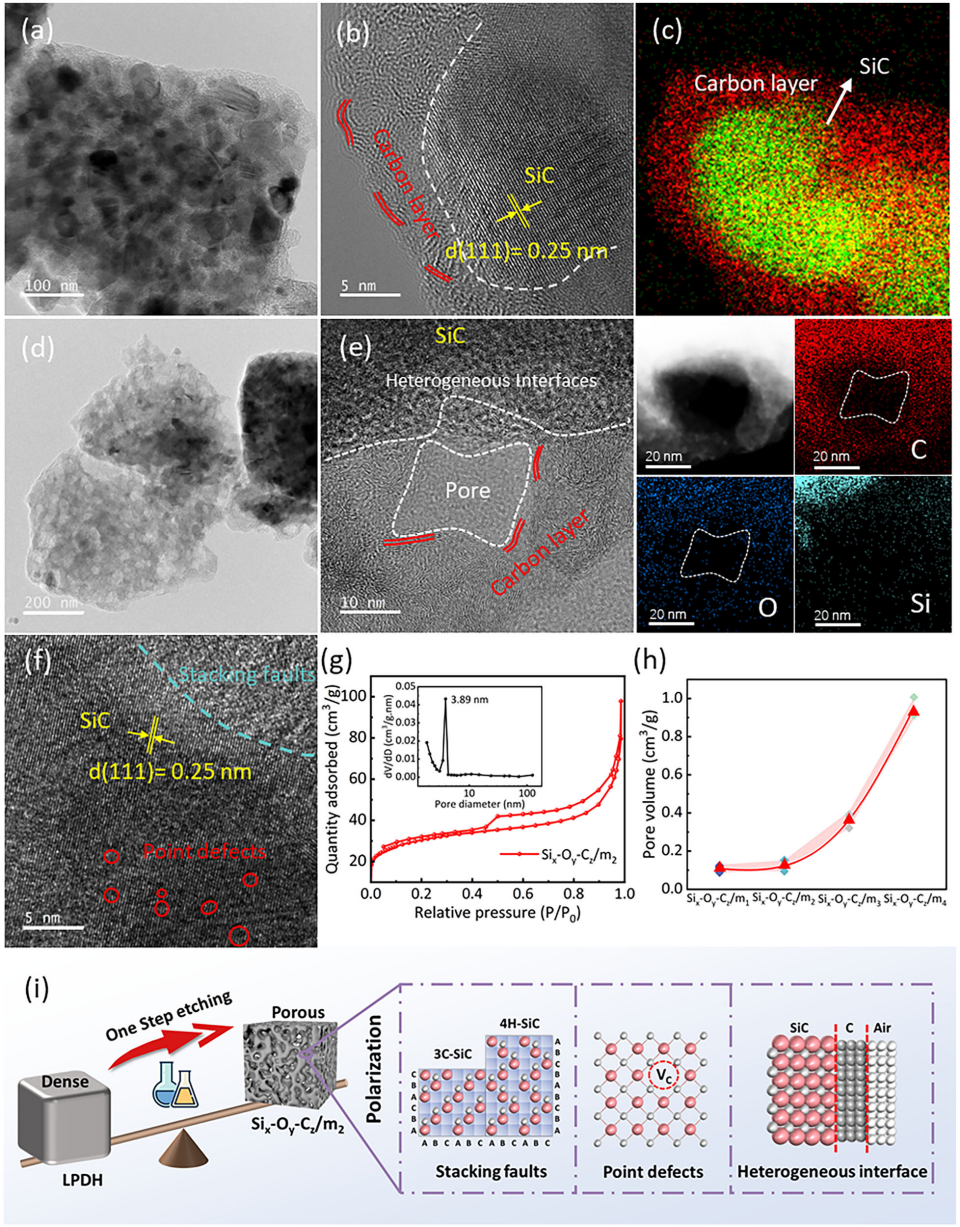
a–c) TEM, HRTEM, and EDS images of LPDH. d–f) TEM, HRTEM, and EDS images of Si_x_‐O_y_‐C_z_/m_2_. g) Nitrogen physisorption isotherms and pore size distribution of Si_x_‐O_y_‐C_z_/m_2_. h) Batch‐to‐batch reproducibility of pore volume in Si_x_‐O_y_‐C_z_ based on BET analysis. The red triangles represent average pore volume and colorful diamonds indicate all experimental results. i) Schematic diagram of dipolar polarization in Si_x_‐O_y_‐C_z_/m_2_.

The microscopic morphology of the synthesized Si_x_‐O_y_‐C_z_ multiphase ceramics was examined using transmission electron microscopy (TEM) and high‐resolution transmission electron microscopy (HRTEM), as presented in **Figure** **3**. Figure 3a,b clearly reveal crystalline β‐SiC particles characterized by a distinct interplanar spacing of ≈0.25 nm, corresponding to the (111) facet of β‐SiC. These crystalline SiC particles are surrounded by an interconnected, relaxed nanocluster or network structure. Energy dispersive spectroscopy (EDS) analysis (Figure 3c) confirms that this network is predominantly composed of carbon. This carbon layer surrounding the SiC crystals plays a key role in facilitating electron migration and accumulation, which is critical for enhancing both interfacial polarization loss and conduction loss, essential for effective microwave absorption.

Figure 3d exhibits the TEM image of the Si_x_‐O_y_‐C_z_/m_2_ multiphase material, which displays a marked morphological contrast compared to the dense structure observed in LPDH. Specifically, a sponge‐like porous architecture is evident, with no discernible SiC particles, highlighting the structural transformation induced by the one‐step etching process. The corresponding HRTEM image (Figure 3e) further confirms the coexistence of SiC, carbon, and pore phases within the hierarchical framework, giving rise to numerous heterogeneous interfaces. These interfaces are crucial in increasing interfacial polarization loss, a key mechanism for effective electromagnetic wave dissipation. Elemental mapping results confirm the presence of the pore phase, as evidenced by the local absence of C, O, and Si signals, supporting the successful incorporation of hierarchical porosity. Moreover, Figure 3f reveals a high density of structure defects – such as stacking faults and point defects – which are known to generate high‐energy unsaturated sites. Stacking faults originate from deviations in the periodic stacking sequence of Si─C bilayers along the c‐axis, resulting in the coexistence of different SiC polytypes such as the cubic structure (3C‐SiC) and hexagonal structure (4H‐SiC) (Figure S5, Supporting Information). These structural inconsistencies lead to lattice deformation, breaking local charge neutrality, and promoting charge carrier migration. Point defects (vacancies, denoted as Vc) are attributed to selective atomic removal induced by alkali etching, and they further enhance interfacial polarization. These sites promote the formation of localized electric dipoles, thereby amplifying dielectric polarization and facilitating electromagnetic energy dissipation. Si_x_‐O_y_‐C_z_/m_1_ exhibits similar structural characteristics to Si_x_‐O_y_‐C_z_/m_2_. (Figure S6, Supporting Information)

The porous structure of the Si_x_‐O_y_‐C_z_ multiphase material was characterized via nitrogen adsorption‐desorption isotherms, which show typical type IV curves with H3 or H4 hysteresis loops (Figure 3g; Figure S7, Supporting Information), confirming the presence of hierarchical porosity. In detail, the rapid increase in nitrogen adsorption at P/P_0_<0.1 indicates the formation of abundant micropores (Figure S8, Supporting Information); the small hysteresis loops detected at P/P_0_ at 0.2–0.8 suggest the presence of mesopores, while the slight rise in nitrogen adsorption at 0.9<P/P_0_<1 reveals a small proportion of macropores. As the pore density increases, the peak at 3.89 nm becomes more prominent, along with additional peaks at tens of nanometers, reflecting a shift toward larger average pore size. To clarify the origin of the hierarchical porosity, a control sample (LPDH‐800) was prepared via pyrolysis without chemical etching. BET and TEM analyses (Figure S9, Supporting Information) show that pyrolyzed‐only samples remain dense and non‐porous, confirming that hierarchical porosity originates from the chemical activation step. BET analysis results of several independent synthesis batches (Figure 3h) show negligible variation in pore volume among different batches, confirming the high reproducibility of the hierarchical porosity control in our synthesis process. Additionally, as shown in Figure S10 (Supporting Information), both the specific surface area and pore volume increase progressively with pore density, which induces abundant heterogeneous interfaces and defects, such as SiC‐C and C‐pores interfaces. Due to significant differences in electronegativity and permittivity across these interfaces, interfacial charge redistribution and accumulation readily occur under an alternating electromagnetic field. This charge separation contributes to interfacial polarization loss, which has been widely reported to enhance EMW attenuation.^[^ ^54^, ^55^, ^56^ ^]^ These factors collectively intensify interfacial polarization, thereby reinforcing the material's electromagnetic wave absorption capability. The schematic in Figure 3i vividly illustrates how the one‐step etching strategy transforms the initially dense ceramics into porous composites rich in polarization‐active sites, including stacking faults, point defects, and heterogeneous interfaces.

**Figure 4:**
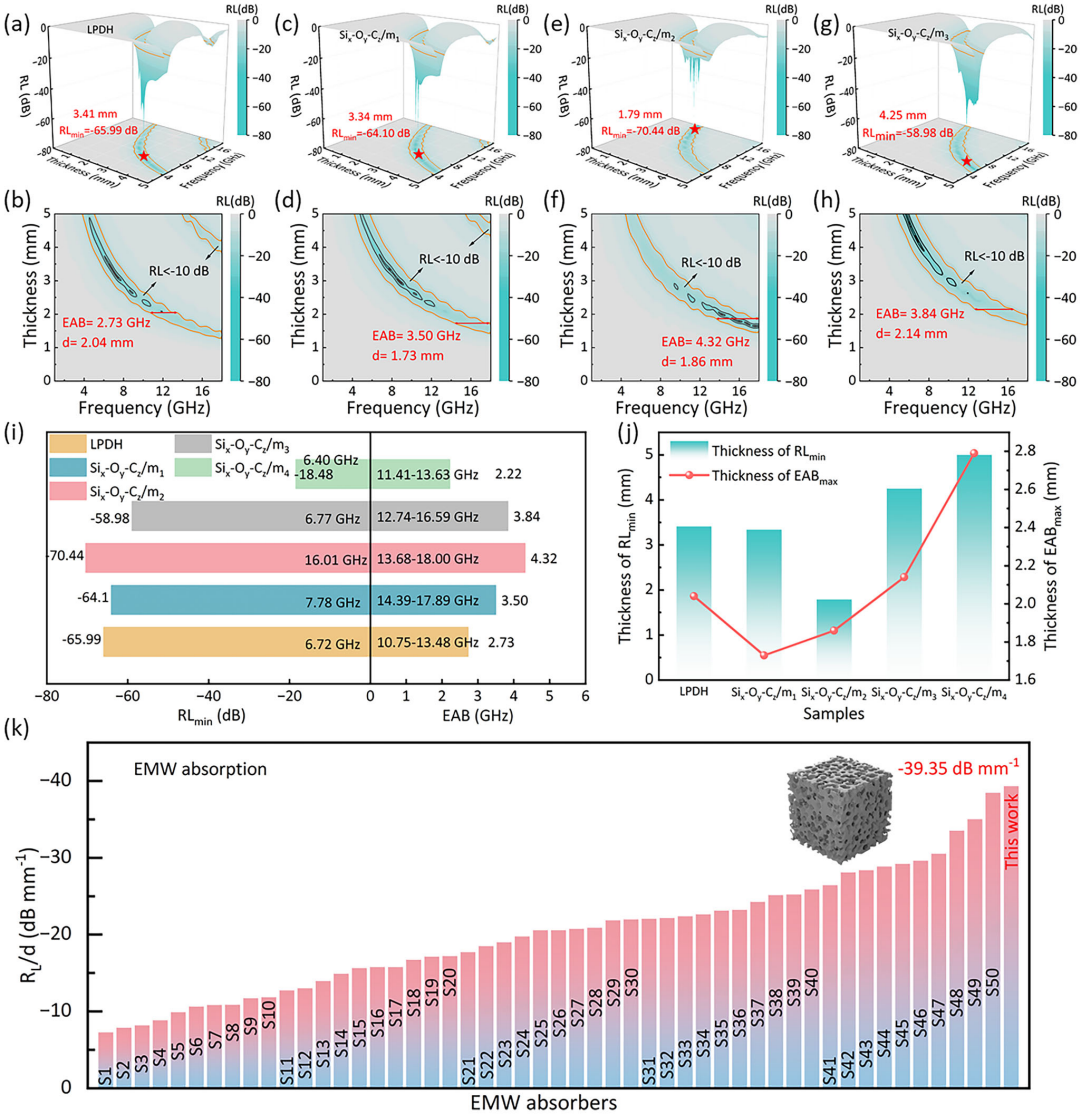
3D and 2D RL patterns of a,b) LPDH, c,d) Si_x_‐O_y_‐C_z_/m_1_, e,f) Si_x_‐O_y_‐C_z_/m_2_, g,h) Si_x_‐O_y_‐C_z_/m_3_. i) Comparison of RL_min_ values and EABs. j) Thickness ranges corresponding to the optimal absorption bandwidth and RL_min_. k) The R_L_/d value of Si_x_‐O_y_‐C_z_/m_2_ was compared with materials listed in references. (see Table S1, Supporting Information for details).

**Figure** **4** illustrates the micromagnetic absorption performance of the Si_x_‐O_y_‐C_z_ multiphase materials with tailored hierarchical porosity. The precursor LPDH exhibits a minimum reflection loss (RL_min_) of −65.99 dB at the thickness of 3.41 mm, with an effective absorption bandwidth (EAB) of 2.73 GHz over 10.75–1.48 GHz (Figure 4a,b). This performance is attributed to its high carbon content and well‐ordered β‐SiC crystalline structure, which facilitates conduction and polarization loss. Upon introducing porosity via controlled one‐step etching activation, the absorption performance is further enhanced due to improved impedance matching and the formation of heterogeneous interfaces. Notably, Si_x_‐O_y_‐C_z_/m_1_ exhibits an RL_min_ of −64.10 dB and an EAB of 3.5 GHz, while Si_x_‐O_y_‐C_z_/m_2_ achieves a superior RL_min_ of −70.44 dB at a reduced matching thickness of only 1.79 mm and a broadened EAB of 4.32 GHz (Figure 4c–f). The obvious improvement in EMW attenuation is closely associated with increased defect density and heterogeneous interfaces, as demonstrated by Raman spectra (Figure 2a), multiphase coexistence in XRD (Figure 2b), the schematically illustrated interfaces in Figure 2g, and TEM results in Figure 3d–f and Figure S6 (Supporting Information). However, excessive pore introduction, as seen in Si_x_‐O_y_‐C_z_/m_3_ and Si_x_‐O_y_‐C_z_/m_4_, leads to deteriorated performance (RL_min_ of −58.98 and −18.48 dB, respectively (Figure 4g,h; Figure S11, Supporting Information)) due to over‐enhanced conductivity and insufficient dielectric loss, as the content of SiOC and SiC phases decreases. Overall, these results underscore that optimizing the phase composition and pore structure is essential for achieving balanced impedance matching and enhanced microwave absorption, with Si_x_‐O_y_‐C_z_/m_2_ emerging as the optimal configuration.

Figure 4i offers a concise overview of the minimum reflection loss and effective absorption bandwidth, consolidating the performance trends observed in Figure 4a–h and Figure S11 (Supporting Information). Notably, Si_x_‐O_y_‐C_z_/m_2_ displays the lowest RL_min_ and the broadest EAB at higher frequencies, underscoring its superior absorption performance. This result highlights the synergy between a moderately increased pore phase – facilitating interfacial polarization and improved impedance matching – and the retention of essential SiC/SiOC phases. Figure 4j shows that the optimal matching thickness for maximum absorption initially decreases with pore introduction, then increases as pore volume further rises. This trend reflects the evolving phase composition: initially, the introduction of pores enhances impedance matching and dielectric properties by facilitating EMW penetration; however, excessive pore formation leads to a gradual loss of SiOC and SiC phases (as seen in Figure 2b), resulting in higher conductivity and insufficient dielectric loss. Thus, precise control over the phase composition is essential to balance conduction and polarization losses, ultimately optimizing microwave absorption performance.

These results demonstrate a strong structure‐property relationship governed by hierarchical porosity. Introducing pores reduces bulk density and activates multiple EMW attenuation mechanisms, including: 1) enhanced interfacial polarization at heterogeneous boundaries (e.g., SiC‐C, C‐pore), 2) improved impedance matching for better EMW penetration, and 3) increased defect density that promotes dipolar polarization and scattering loss. However, excessive porosity deteriorates the SiC/SiOC phases, raising conductivity and weakening absorption. As shown in Figure 4, Si_x_‐O_y_‐C_z_/m_2_, with moderate pore density, offers the best balance between impedance matching and dielectric loss, highlighting the importance of well‐regulated porosity in optimizing EMW absorption in multiphase ceramics.

Additionally, RL_min_‐to‐thickness ratio (R_L_/d) was calculated to quantitatively assess thickness‐normalized loss performance (Figure 4k). A higher R_L_/d value indicates stronger absorption capability at lower thickness, demonstrating the competitive EMW absorption performance of Si_x_‐O_y_‐C_z_/m_2_ in terms of thinness and efficiency. Compared with recently reported ceramic‐based absorbers, Si_x_‐O_y_‐C_z_/m_2_ exhibits one of the highest R_L_/d values. To clarify the performance origin, normalized input impedance (Z = Z_in_/Z_0_) was used to assess impedance matching. As shown in Figure S12 (Supporting Information), Si_x_‐O_y_‐C_z_/m_2_ shows lower |Z − 1| values over a broad frequency range and at thin thicknesses, indicating improved impedance matching and greater EMW penetration. This balance of impedance matching and dielectric loss‐enabled by hierarchical porosity and multiphase composition‐accounts for its superior absorption.

**Figure 5:**
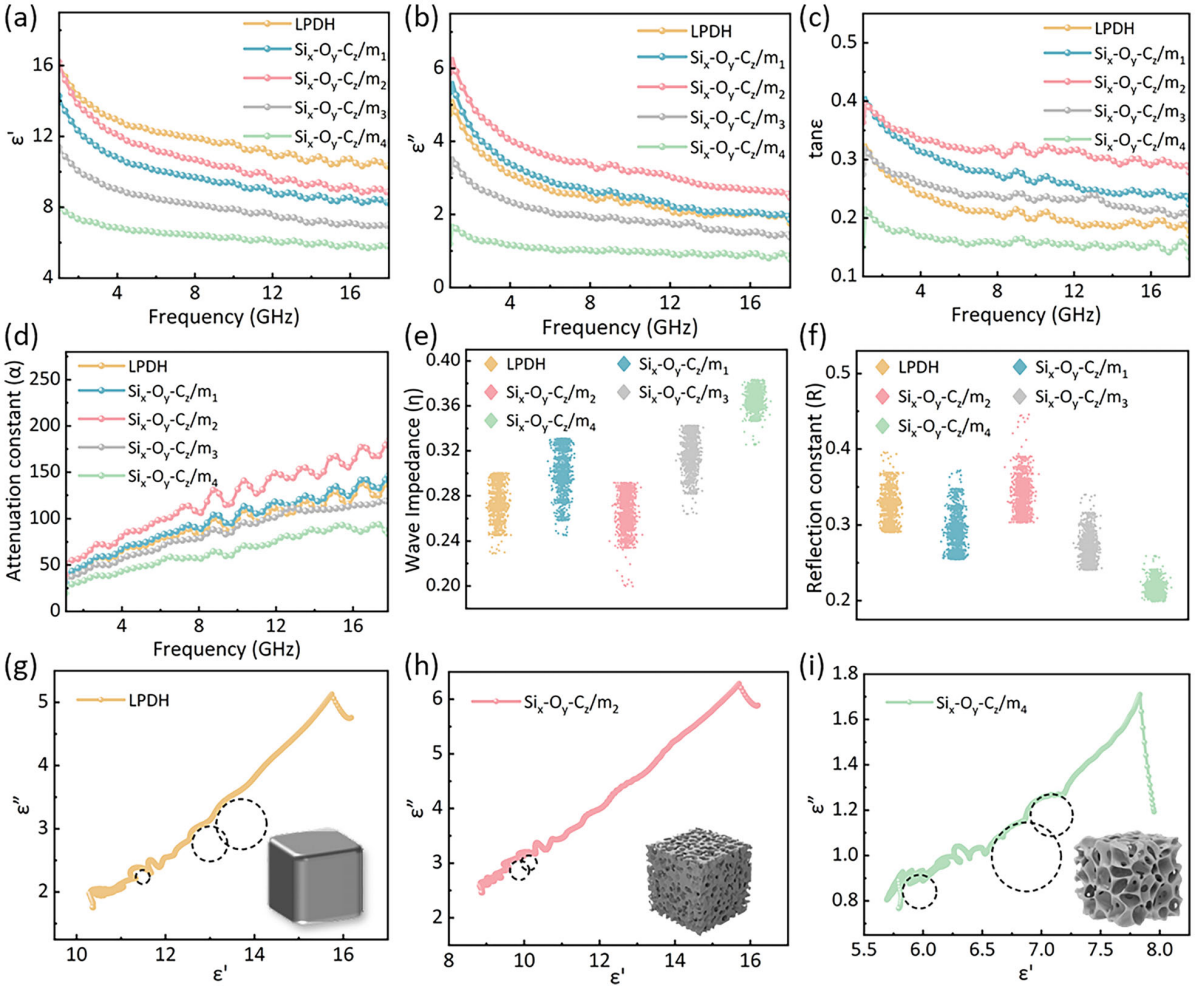
Frequency‐dependent a) ε', b) ε'', c) tanε, d) α, e) Wave impedance, f) Reflection constant, g–i) Cole‐Cole plots of LPDH, Si_x_‐O_y_‐C_z_/m_2_, and Si_x_‐O_y_‐C_z_/m_4_.

To elucidate the underlying mechanisms, the real (ε') and the imaginary (ε'') parts of the complex permittivity, along with the dielectric loss tangent (tanσ), were measured via the waveguide method (**Figure** **5**). Here, ε' represents the capacity to store electromagnetic energy, ε'' indicates energy dissipation, and tanσ (ε'/ε'') reflects the overall attenuation capability of the material. As shown in Figure 5a,b, ε' and ε'' for all samples generally decrease with increasing frequency, attributable to stronger dispersion effects and dipole relaxation at higher frequencies.^[^ ^57^ ^]^ Upon introducing the pore phase, ε' initially declines, likely because certain SiC‐carbon interfaces are disrupted, reducing space charge accumulation. With further pore formation, ε' rises again, presumably due to new pore‐carbon interfaces that promote localized polarization, before ultimately dropping when excessive pore channels compromise the structural continuity. Conversely, ε'' increases at first, driven by moderate conductivity enhancement and interfacial polarization, then drops below that of LPDH as porosity becomes excessive, leading to over‐enhanced conductivity and insufficient dielectric loss. The tanσ (Figure 5c) underscores these observations: Si_x_‐O_y_‐C_z_/m_2_ exhibits the highest tanσ, highlighting its strong EMW dissipation capability. These findings are consistent with the multiphase structural evolution described, demonstrating that moderate pore formation boosts conduction and interfacial polarization, whereas excessive pore volume undermines the delicate balance required for optimal EMW absorption. In addition to permittivity, the attenuation constant (α) as another crucial parameter is evaluated. As shown in Figure 5d, α increases steadily with frequency, indicating enhanced energy dissipation at higher frequencies. Notably, Si_x_‐O_y_‐C_z_/m_2_ exhibits the highest α values, especially in the high‐frequency range, underscoring its superior attenuation capability.

The intrinsic impedance matching properties, characterized by the wave impedance (η), are also critical for effective absorption. In general, an η value greater than 0.3 facilitates the entry of EMWs into the material.^[^ ^58^ ^]^ Figure 5e shows an S‐shaped variation in η, reflecting the complex evolution of the phase composition. Samples with pore phases generally display higher η values compared to pristine LPDH, indicating improved impedance matching. In Figure 5f, an inverse trend between wave impedance and the reflection constant (R) is observed; although LPDH and Si_x_‐O_y_‐C_z_/m_2_ exhibit higher reflection coefficients, Si_x_‐O_y_‐C_z_/m_2_ still achieves the best overall EMW absorption performance. This is because the EMW absorption efficacy is synergistically determined by both impedance matching and loss strength. Once a sufficient level of impedance matching is reached, further enhancement of dielectric loss (loss strength) becomes the dominant factor. Consequently, the optimized pore structure in Si_x_‐O_y_‐C_z_/m_2_ not only improves impedance matching but also significantly boosts dielectric loss, leading to superior overall absorption performance.

To further clarify the dielectric loss mechanism of these materials, Cole–Cole curves were plotted based on the following Debye relaxation equation:

(2) $${\left(\varepsilon^{\prime }-\;\frac{\varepsilon_{s}+\varepsilon_{\infty }}{2}\right)}^{2}+\;{\left(\varepsilon^{\prime \prime }\right)}^{2}={\left(\frac{\varepsilon_{s}+\varepsilon_{\infty }}{2}\right)}^{2}$$

where  ε_*s*_ and ε_∞_ are the static permittivity and high‐frequency permittivity, respectively. According to Debye theory, semicircular features in the Cole–Cole plots represent polarization relaxation loss, whereas linear segments indicate conduction loss. As illustrated in Figure 5g, the Cole–Cole curve of pristine LPDH primarily exhibits a linear characteristic with minimal semicircular features, suggesting conduction loss plays a dominant role. For Si_x_‐O_y_‐C_z_/m_1_ and Si_x_‐O_y_‐C_z_/m_2_ (Figure S13a, Supporting Information; Figure 5h), the presence of semicircles is even more suppressed, indicating that conduction loss significantly overshadows polarization relaxation contributions due to improved conductivity. However, with further increased pore volume (Si_x_‐O_y_‐C_z_/m_3_ and Si_x_‐O_y_‐C_z_/m_4_, Figure S13b, Supporting Information; Figure 5i), numerous heterogeneous interfaces and defect sites emerge prominently, enhancing polarization losses again. Consequently, these samples exhibit multiple pronounced semicircles combined with linear segments, highlighting that polarization and conduction losses synergistically contribute to their overall dielectric loss and EMW absorption performance.

**Figure 6:**
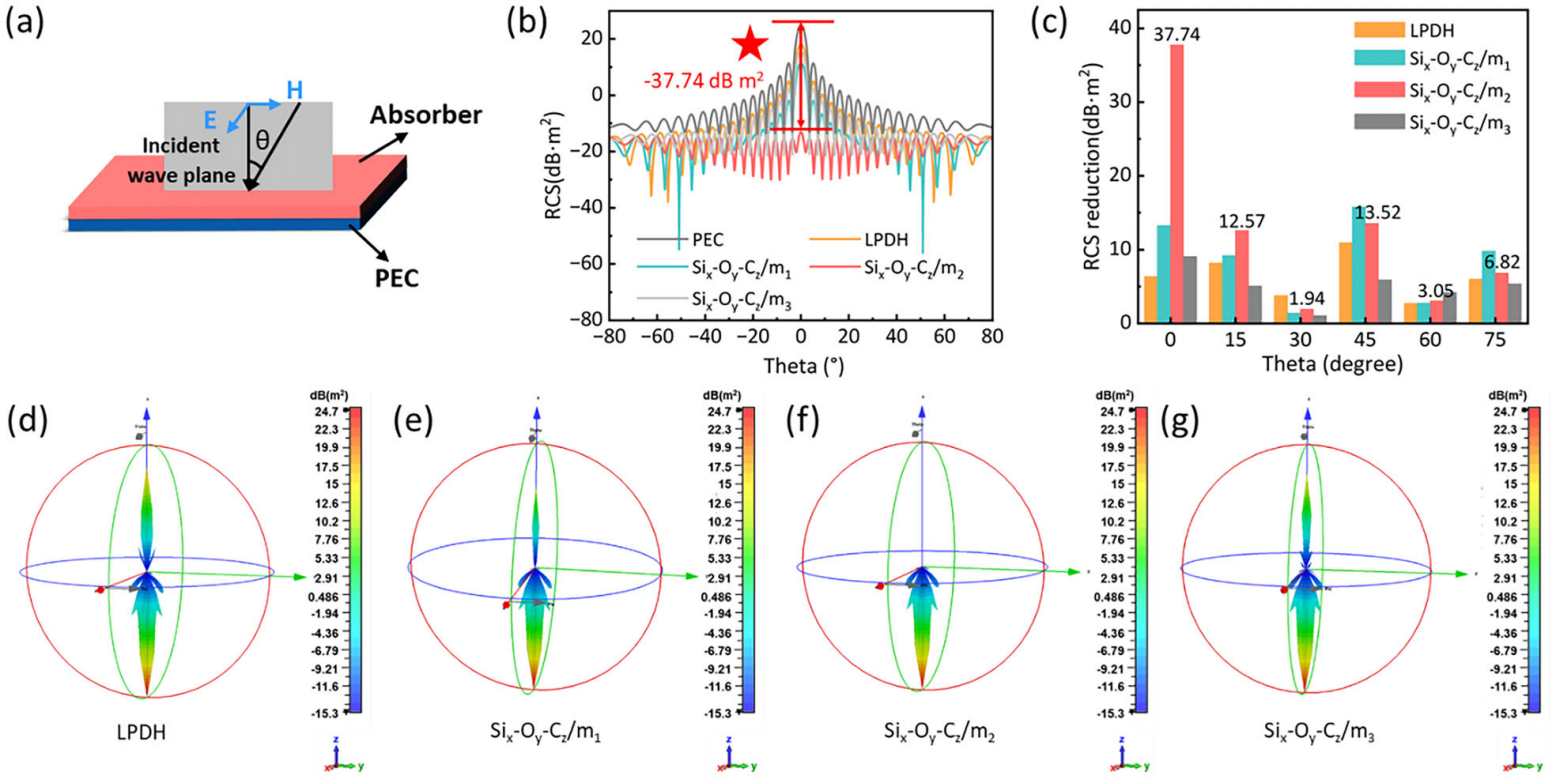
a) Schematic diagram of the model used in CST simulation. b) RCS values and c) RCS reduction values at specific angles are obtained by CST simulation. d–g) 3D radar wave scattering signals of LPDH, Si_x_‐O_y_‐C_z_/m_1_, Si_x_‐O_y_‐C_z_/m_2_, Si_x_‐O_y_‐C_z_/m_3_.

To evaluate the stealth potential of the Si_x_‐O_y_‐C_z_ material in practical radar applications, the radar cross‐section (RCS) of perfect electric conductor (PEC) plate, both bare and coated with LDPH or Si_x_‐O_y_‐C_z_, was simulated using the Computer Simulation Technology (CST) Studio Suite (**Figure** **6**).^[^ ^59^ ^]^ The simulation model is depicted in Figure 6a. As shown in Figure 6b, the RCS values of PEC, LPDH, Si_x_‐O_y_‐C_z_/m_1_, Si_x_‐O_y_‐C_z_/m_2_, and Si_x_‐O_y_‐C_z_/m_3_ were calculated across a wide angular range. Notably, Si_x_‐O_y_‐C_z_/m_2_ maintains an RCS value below −15 dB m^2^ across −80^°^ to 80^°^, indicating outstanding angular stability in stealth performance. At the normal incidence (0^°^), Si_x_‐O_y_‐C_z_/m_2_ achieves a maximum RCS reduction of 37.74 dB m^2^ (Figure 6c), significantly surpassing the other samples. The 3D scattering field distributions (Figure 6d–g; Figure S14, Supporting Information) further visualize this effect, which exhibits dramatically attenuated backscattering compared to the PEC baseline. These results highlight the excellent wave absorption and scattering suppression capabilities of Si_x_‐O_y_‐C_z_/m_2_, demonstrating its strong potential for practical electromagnetic stealth applications.

**Figure 7:**
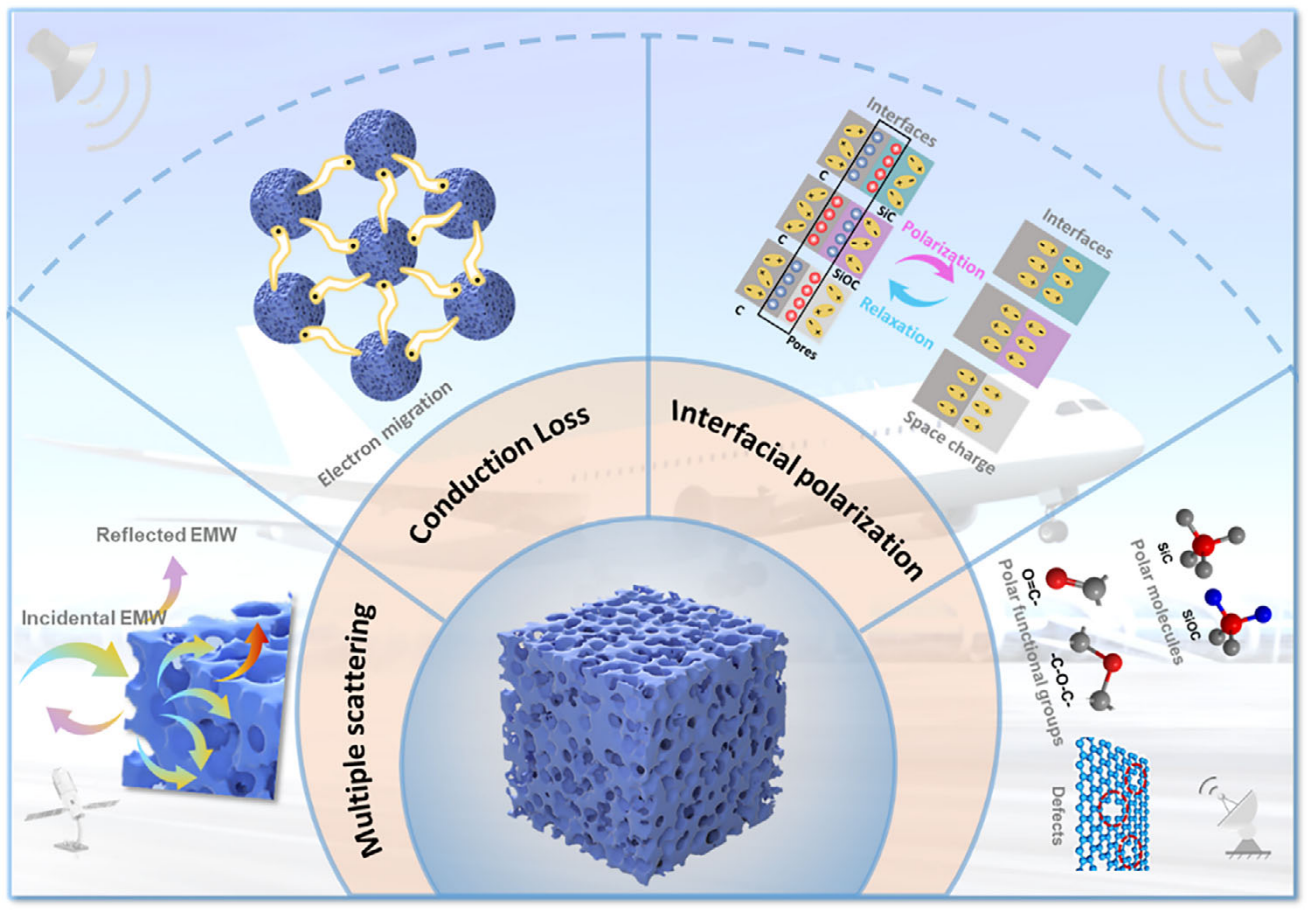
Schematic illustration of the EMW absorption mechanism of multiphase Si‐based ceramics.

The EMW absorption mechanisms of the novel multiphase Si_x_‐O_y_‐C_z_ material are schematically illustrated in **Figure** **7**. First, the abundant carbon phase derived from phenyl groups in DVB establishes conductive pathways for electron migration and hopping, significantly contributing to conduction loss. Meanwhile, structural defects and pores within this carbon phase create abundant dipole centers, effectively enhancing dipole polarization loss.^[^ ^60^ ^]^ Second, heterogeneous interfaces among carbon, SiC, SiOC, and pore phases facilitate electron accumulation under alternating electromagnetic fields, strongly promoting interfacial polarization. The presence of SiC and SiOC phases, characterized by relatively lower dielectric constants, further optimizes impedance matching, enabling more EMWs to penetrate the material. Third, the introduction of porous structure reduces the effective dielectric constant, thereby improving impedance matching, and simultaneously provides additional scattering centers and interfaces to amplify EMW attenuation through scattering and interfacial polarization. Additionally, the SiOC phase serves dual roles by acting as polarization centers and contributing to interfacial polarization at its boundaries.^[^ ^61^ ^]^ The mechanism shown in Figure 7 echoes the synthetic and structural pathways depicted in Figure 1. The abundant heterogeneous interfaces between crystalline SiC, amorphous SiOC, and carbon phases, coupled with the hierarchical porosity and defect engineering, jointly contribute to conduction loss, interfacial polarization, and multiple reflections. This multiphase synergy results in enhanced attenuation across a wide frequency band. To assess industrial applicability, the thermal, chemical, and moisture stability of Si_x_‐O_y_‐C_z_ was evaluated. After 7 days in acid (pH 1), alkali (pH 13), salt (5 wt.% NaCl), and deionized water, the porous structure remained intact (Figure S15, Supporting Information). Pyrolysis at 1200 °C under Ar also preserved porosity (Figure S16, Supporting Information), confirming high structural and thermal stability. Moreover, the one‐step, template‐free synthesis uses low‐cost precursors and mild conditions, enabling gram‐to‐hundred‐gram scale‐up. These advantages underscore the method's industrial potential for scalable EMW absorber production. Ultimately, the synergistic interaction among these phases and mechanisms achieves superior microwave absorption performance, highlighting the effectiveness of this multiphase design strategy and providing new insights into the development of advanced ceramic absorbers.

## 3 Conclusion

In summary, we report a novel multiphase Si‐based ceramic material with hierarchical porosity, denoted as Si_x_‐O_y_‐C_z_, via a simple, one‐step etching activation of carbon‐rich polycarbosilane ceramics. This method enables the simultaneous formation of hierarchical pore structures and multiphase compositions, including free carbon, crystalline SiC, amorphous SiOC, and engineered pore domains, without requiring complex templating or post‐treatment procedures. The in situ generation of heterogeneous interfaces, stacking faults, and abundant dipolar sites collectively enhances conduction loss, interfacial polarization, dipolar relaxation, and multiple internal scatterings, leading to excellent EMW attenuation capabilities.

Among the synthesized variants, Si_x_‐O_y_‐C_z_/m_2_ demonstrates the best performance, achieving a minimum reflection loss of −70.44 dB at a thickness of only 1.79 mm and an effective absorption bandwidth of 4.32 GHz at a matching thickness of 1.86mm. The optimized structure balances impedance matching with dielectric loss, and the favorable radar cross‐section suppression across wide angles further validates its stealth capability in real‐world scenarios. Importantly, the demonstrated chemical, thermal, and moisture stability, along with the reproducibility and simplicity of the synthesis route, make it highly feasible for scalable production. This work offers new insights into the synergistic design of multiphase ceramics and porous architectures and establishes a promising pathway for developing broadband and high‐efficiency EMW absorbing materials for advanced electromagnetic compatibility and stealth technologies.

## 4 Experimental Section

### Materials

Strong alkali, such as sodium hydroxide (NaOH, 95%) and potassium hydroxide (KOH, 95%), and divinylbenzene (DVB, C_10_H_10_, 80%) were all purchased from Aladdin (China). Dimethylbenzene (C_8_H_10_, 99%) was obtained from Macklin (China). Liquid polycarbosilane (LPCS, Me‐(CH = CH)_x_‐(CHMe‐SiHMe)_y_‐Me) was provided by Suzhou CeraFil Co., Ltd. Platinum complex ([Pt]:3000 ppm, Zhengzhou Alfa Chemical Co., Ltd., China) was selected as the catalyst for hydrosilylation of LPCS and DVB. All chemicals were used without any purification.

### Synthesis of Carbon‐Rich Si‐Based Ceramics

First, 200 mL of dimethylbenzene was added to a 500 mL three‐necked flask and then heated to 130 °C. To initiate the reaction, 20 g LPCS, 20 g DVB, and 0.36 g platinum complex were sequentially introduced to the solution and allowed to react for 6 h. Then the solvent was evaporated using a rotary evaporator at 90 °C for 2 h and 160 °C for 1 h to remove dimethylbenzene, and the precipitate was denoted as LPD. The LPD samples underwent a post‐synthesis thermal treatment to enhance their properties. The procedure involved heating the samples to 300 °C for 2 h under an argon gas atmosphere, with a controlled temperature increase of 5 °C min^−1^. Subsequently, the samples were heated to 1600 °C for 2 h at a 5 °C min^−1^ ramp rate. After carrying out these operations, experimental intermediate products, carbon‐rich Si‐based ceramics (LPDH), were obtained.

### Preparation of Multiphase Si‐Based Ceramics

A mixture of LPDH and strong alkali, such as NaOH, and KOH, with different ratios, was ball‐ground for 30 min to blend uniformly. Subsequently, the mixture was placed in a nickel boat and heated to 800 °C for 2 h under argon flow at a 5 °C min^−1^ ramp rate in a tube furnace. The products obtained were washed with copious amounts of deionized water several times until the filtrate became neutral. The isolated products were subjected to a drying process under vacuum at 60 °C, and the target products, multiphase Si‐based ceramics, were obtained. The materials obtained after the drying process were defined as Si_x_‐O_y_‐C_z_/m where m represents the alkali‐to‐LPDH ratio (0.5, 1, 2, and 4 for Si_x_‐O_y_‐C_z_/m_1_, Si_x_‐O_y_‐C_z_/m_2_, Si_x_‐O_y_‐C_z_/m_3_ and Si_x_‐O_y_‐C_z_/m_4_, respectively). These specimens were then meticulously analyzed to determine their phase compositions and microwave absorption performances.

### Characterization

Fourier‐transform infrared (FT‐IR) spectra were acquired using a Bruker TENSOR27 spectrometer, employing KBr discs to analyze the samples. Carbon (^13^C) nuclear magnetic resonance (NMR) spectra were recorded on a Bruker Avance Neo 400WB, with tetramethylsilane (TMS) serving as the external standard for chemical shift referencing. The crystalline structure and phase purity of the samples were investigated using X‐ray diffraction (XRD) on a Smartlab‐9 system from Rigaku. Raman spectra were obtained using an inVia confocal Raman microscope spectrometer from Renishaw, with a laser excitation wavelength of 532 nm. The surface chemical states of the samples were probed using X‐ray photoelectron spectroscopy (XPS) on an AXIS SUPRA+ instrument from Shimadzu. The microstructure and morphology of the synthesized samples were examined using a JEM‐F200 transmission electron microscope (TEM) from JEOL. This high‐resolution imaging allowed for the detailed observation of the samples at the nanometer scale. The nitrogen physisorption isotherms and pore size distribution in the nitrogen atmosphere were analyzed using an N_2_ adsorption‐desorption analyzer (Micromeritics ASAP 2020 PlusHD88).

### Microwave Absorption Measurement

An Agilent E5071C vector network analyzer was used to measure the electromagnetic parameters of the samples in the microwave frequency range (1–18 GHz) and evaluate their EMW absorption performance. The homogeneous mixture of the sample and paraffin, the mass ratio of which changes from 5:5 to 2:8 according to the conductivity of the sample, was pressed into a test ring with an inner diameter of 3.04 mm and an outer diameter of 7.00 mm. Microwave absorbing performance was evaluated by the reflection loss, which can be calculated based on the transmission line theory:^[^ ^62^ ^]^

(3) $$R_{L}=20\log_{10}\left|(Z_{\mathrm{in}}-Z_{0})/(Z_{\mathrm{in}}+Z_{0})\right|$$

(4) $$Z_{in}=Z_{0}{\left(\mu_{r}/\varepsilon_{r}\right)}^{1/2}\tanh\;\left[j\left(2\pi fd\right){\left(\mu_{r}\varepsilon_{r}\right)}^{1/2}/c\right]$$

where the Z_0_ and Z_in_ represent the free‐space impedance and input impedance of the sample, respectively, ɛ_r_ and μ_r_ are the complex permittivity (ε_r_ = ε' − jε'') and permeability (μ_r_ = μ' − jμ''), respectively, f is the frequency of microwave, c is the light velocity, and d is the absorber thickness.

### RCS Simulation

The RCS value can be calculated as follows. Radar cross‐section (RCS) simulations were performed using CST Studio Suite 2019 to evaluate the stealth performance of the absorbers. A model of a double‐layer metal backing with dimensions of 300 × 300 mm^2^ was constructed, consisting of an upper absorber layer (thickness = 1.79 mm) and a lower perfect electrical conductor (PEC) substrate. The simulation uses fully open boundary conditions and the monitoring frequency was fixed at 16.01 GHz. The angle of incidence of the electromagnetic wave varies from −180° to 180° to assess the effect of the angle on the RCS.

The RCS value was calculated using the following equation:

(5) $$\mathrm{RCS}\;\left(\mathrm{dB}\;m^{2}\right)=10\log{\left\{\left(4\pi S\;/\;\lambda^{2}\right)\left(\left|E_{S}\;/\;E_{i}\right|\right)\right\}}^{2}$$

where S is the surface area of the simulation target, λ is the wavelength of the incident electromagnetic wave, E_s_ and E_i_ are the scattered and incident electric field intensities, respectively.

## Conflict of Interest

The authors declare no conflict of interest.

## Supporting information



Supporting Information

## Data Availability

The data that support the findings of this study are available from the corresponding author upon reasonable request.

## References

[1] L. Zhou , P. Hu , M. Bai , N. Leng , B. Cai , H.‐L. Peng , P.‐Y. Zhao , Y. Guo , M. He , G.‐S. Wang , J. Gu , Adv. Mater. 2025, 37, 2418321. 10.1002/adma.202418321 39726342

[2] X. Zeng , L. Wu , X. Yang , Z. Wu , X. Xu , K. Pei , W. You , H.‐W. Cheng , R. Che , Adv. Funct. Mater. 2025, 35, 2502671.

[3] Y. Li , W. Zhai , G. Wang , Y. Deng , S. Du , S. Li , C. Fu , D. Lan , S. Zhang , X. Wang , X. Yu , G. Wu , Nano Res. 2025, 18, 94907470.

[4] R. Wang , Q. Liu , J. Wei , C. Zhu , Y. Wang , A. Yu , W. Wang , J. Zou , J. Xie , Z. Fu , J. Mater. Sci. Technol. 2025, 210, 195.

[5] S. Zhang , J. Zheng , D. Lan , Z. Gao , X. Liang , Q. Tian , Z. Zhao , G. Wu , Adv. Funct. Mater. 2025, 35, 2413884.

[6] J. Xiao , B. Zhan , M. He , X. Qi , Y. Zhang , H. Guo , Y. Qu , W. Zhong , J. Gu , Adv. Funct. Mater. 2025, 35, 2419266.

[7] X. Zeng , C. Zhao , X. Jiang , R. Yu , R. Che , Small 2023, 19, 2303393. 10.1002/smll.202303393 37291740

[8] B. Hao , Y. Zhang , H. Si , Z. Jiang , C. Li , Y. Zhang , J. Zhang , C. Gong , Adv. Funct. Mater., 35, 2423897.

[9] X. Zeng , T. Nie , C. Zhao , Y. Gao , X. Liu , Adv. Sci. 2024, 11, 2403723. 10.1002/advs.202403723 PMC11425237 39013079

[10] Q. Wen , Z. Yu , R. Riedel , Prog. Mater Sci. 2020, 109, 100623.

[11] Q. Wen , F. Qu , Z. Yu , M. Graczyk‐Zajac , X. Xiong , R. Riedel , J. Adv. Ceram. 2022, 11, 197.

[12] W. Li , Z. Yu , Q. Wen , Y. Feng , B. Fan , R. Zhang , R. Riedel , Int. Mater. Rev. 2022, 68, 487.

[13] X. Zeng , E. Li , G. Xia , N. Xie , Z.‐Y. Shen , M. Moskovits , R. Yu , J. Eur. Ceram. Soc. 2021, 41, 7381.

[14] J. Huang , X. Zeng , X. Jiang , X. Deng , Q. Fu , Y. Xie , Y. Gao , Chem. Eng. J. 2025, 503, 158520.

[15] J. Bai , S. Huang , X. Yao , X. Liu , Z. Huang , Chem. Eng. J. 2023, 469, 143809.

[16] Q. Li , X. Yin , W. Duan , L. Kong , B. Hao , F. Ye , J. Alloys Compd. 2013, 565, 66.

[17] Y. Huo , K. Zhao , M. Peng , F. Li , Z. Lu , Q. Meng , Y. Tang , J. Alloys Compd. 2022, 891, 162006.

[18] W. Yang , C. Liu , Y. Hou , X. Dai , C. Zhong , Y. Ma , M. Jiang , Chem. Eng. J. 2025, 506, 160189.

[19] S. Wang , H. Gong , M. Z. Ashfaq , D. Qi , X. Yue , Ceram. Int. 2022, 48, 23989.

[20] Q. Chen , D. Li , Z. Yang , D. Jia , Y. Zhou , R. Riedel , T. Zhang , C. Gao , Carbon 2021, 179, 180.

[21] F. Ye , Q. Song , Z. Zhang , W. Li , S. Zhang , X. Yin , Y. Zhou , H. Tao , Y. Liu , L. Cheng , L. Zhang , H. Li , Adv. Funct. Mater. 2018, 28, 1707205.

[22] Z. Xiang , B. Xu , Q. He , Y. Wang , X. Yin , Chem. Eng. J. 2023, 457, 141198.

[23] M. Wei , L. Wang , S. U. Rehman , W. Zhang , S. Shen , B. Peng , Y. Hu , T. Liang , J. Am. Ceram. Soc. 2023, 107, 3290.

[24] Y. Han , H. Guo , H. Qiu , J. Hu , M. He , X. Shi , Y. Zhang , J. Kong , J. Gu , Adv. Funct. Mater. 2025, 35, 2506803.

[25] X.‐Z. Tang , Z. Zhao , Z. Liao , J. Yue , W. Gong , H. Zhang , Y. Wang , Compos. Commun. 2024, 52, 102131.

[26] R. Cao , Y. Qiu , X. Zhao , D. Lan , Y. Lu , Z. Wang , H. Xu , Y. Liu , Diam. Relat. Mater. 2025, 157, 112542.

[27] X. Wang , J. Liu , X. Han , A. Deng , B. Han , Y. Jin , D. Lan , M. Ma , Y. Li , J. Alloys Compd. 2025, 1028, 180631.

[28] L. Ma , Y. Deng , X. Pei , Z. Huang , Q. Huang , Silicon 2025, 17, 851.

[29] M. He , X. Zhong , X. Lu , J. Hu , K. Ruan , H. Guo , Y. Zhang , Y. Guo , J. Gu , Adv. Mater. 2024, 36, 2410186. 10.1002/adma.202410186 39380425

[30] X. You , G. Dai , R. Deng , T. Zhang , L. Song , X. Zhang , Y. Ding , J. Yang , S. Dong , Addit. Manuf. 2022, 55, 102855.

[31] L. Duan , X. Dai , F. Wu , A. Xie , J.‐A. Wu , M. Sun , Y. Xia , Nanomaterials 2021, 11, 3438. 34947787 10.3390/nano11123438 PMC8706827

[32] S. Dong , Y. Chen , C. Hong , J. Alloys Compd. 2020, 838, 155558.

[33] X. Zeng , X. Deng , J. Huang , Y. Gao , H. Lv , Nano Today 2025, 63, 102770.

[34] X. Lan , Z. Wang , Carbon 2020, 170, 517.

[35] J. Jiang , L. Yan , J. Li , C. Zhang , X. Hu , A. Guo , H. Du , J. Liu , Ceram. Int. 2025, 51, 2315.

[36] Q. Cao , H. Qian , T. Liu , N. Li , Y. Huang , D. Jiang , B. Jiang , Chem. Eng. J. 2025, 509, 161486.

[37] T. Xiao , J. Kuang , H. Pu , Q. Zheng , Y. Lu , W. Liu , W. Cao , J. Alloys Compd. 2021, 862, 158032.

[38] J. Xiao , B. Zhan , M. He , X. Qi , X. Gong , J.‐L. Yang , Y. Qu , J. Ding , W. Zhong , J. Gu , Adv. Funct. Mater. 2025, 35, 2316722.

[39] J. Jiang , L. Yan , M. Song , Y. Li , A. Guo , H. Du , J. Liu , Ceram. Int. 2025, 51, 17.

[40] Z. Wang , J. Liu , H. Hao , Q. Jing , S. Yan , J. Guo , Z. Wang , Carbon 2024, 217, 118622.

[41] Z. Wang , Y. Hou , H. Hao , Y. Shuai , Z. Wang , Carbon 2023, 211, 118092.

[42] L. Song , L. Wang , Y. Chen , H. Wu , B. Song , N. Wang , L. Guan , H. Wang , R. Zhang , Y. Zhu , Y. Xia , B. Fan , Small 2024, 20, 2407563. 10.1002/smll.202407563 39420747

[43] S. Lv , H. Luo , Z. Wang , J. Yu , F. Chen , Y. Cheng , X. Li , Surf. Interfaces 2024, 50, 104530.

[44] B. Du , J. Qian , P. Hu , C. He , M. Cai , X. Wang , A. Shui , Carbon 2020, 157, 788.

[45] Y. Ning , X. Zeng , J. Huang , Z. Y. Shen , Y. Gao , R. Che , Adv. Funct. Mater. 2024, 35, 2414838.

[46] X. Zeng , X. Peng , Y. Ning , X. Jiang , R. Yu , X. Zhang , J. Mater. Sci. Technol. 2024, 192, 6.

[47] J. Bai , Z. Xie , P. Zhang , S. Wang , Z. Liu , X. Yao , X. Liu , Z. Huang , Compos. Part B Eng. 2025, 294, 112164.

[48] N. S. Jacobson , Y. G. Gogotsi , M. Yoshimura , J. Mater. Chem. 1995, 5, 595.

[49] G. Yury G , K. G. Nickel , D. Bahloul‐Hourlier , T. Merle‐Mejean , G. E. Khomenkod , K. P. Skjerlie , J. Mater. Chem. 1996, 6, 595.

[50] J. Yang , H. Wu , M. Zhu , W. Ren , Y. Lin , H. Chen , F. Pan , Nano Energy 2017, 33, 453.

[51] Z. Shen , Y. Zu , Y. Chen , J. Gong , C. Sun , J. Mater. Sci. Technol. 2023, 137, 79.

[52] K. Zou , Y. Deng , J. Chen , Y. Qian , Y. Yang , Y. Li , G. Chen , J. Power Sources 2018, 378, 579.

[53] Z. Guo , P. Ren , F. Zhang , H. Duan , Z. Chen , Y. Jin , F. Ren , Z. Li , J. Colloid Interface Sci. 2022, 610, 1077. 34887064 10.1016/j.jcis.2021.11.165

[54] M. Qin , L. Zhang , H. Wu , Adv. Sci. 2022, 9, 2105553. 10.1002/advs.202105553 PMC8981909 35128836

[55] W. Gu , Z. Luo , J. Wang , X. Tan , Z. Tao , P. Zhou , H. Zhang , D. Lan , A. Xia , J. Mater. Sci. Technol. 2026, 243, 102.

[56] M. Shi , Z. Jia , D. Lan , Z. Gao , S. Zhang , G. Wu , Adv. Funct. Mater. 2025, 02261.

[57] L. Chai , Y. Wang , Z. Jia , Z. Liu , S. Zhou , Q. He , H. Du , G. Wu , Chem. Eng. J. 2022, 429, 132547.

[58] D. Fei , Y. Guo , J. Zhou , J. Tao , N. Chen , Z. Yao , X. Tao , L. Duan , J. Liu , B. Ouyang , Mater. Res. Bull. 2024, 173, 112662.

[59] B. Zhan , Y. Qu , X. Qi , J. Ding , J.‐J. Shao , X. Gong , J.‐L. Yang , Y. Chen , Q. Peng , W. Zhong , H. Lv , Nano‐Micro Lett. 2024, 16, 221. 10.1007/s40820-024-01447-9 PMC11183034 38884840

[60] Z. Wang , Z. Jia , J. Ren , L. Wang , D. Lan , S. Zhang , X. Shi , X. Liu , Z. Gao , G. Wu , J. Mater. Sci. Technol. 2025, 235, 81.

[61] Y. Cheng , X. Liu , J. Ren , X. Xu , D. Lan , G. Wu , S. Zhang , Z. Gao , Z. Jia , G. Wu , Carbon 2025, 239, 120325.

[62] Z. Wu , J. Huang , X. Zeng , Soft Sci. 2024, 4, 42.

