# Supplementary material for: Hierarchically Porous Multiphase Si‐Based Ceramics with Synergistic Electromagnetic Wave Absorption Mechanisms
Source: Adv Sci (Weinh). 2025 Aug 14;12(42):e10445. doi: 10.1002/advs.202510445 (PMC12622537; doi:10.1002/advs.202510445)
Supplement: Supplementary file 1 — Supporting Information [file ADVS-12-e10445-s001.docx]

Supporting Information

**Hierarchically Porous Multiphase Si-Based Ceramics with Synergistic Electromagnetic Wave Absorption Mechanisms**

*Jiaojiao Jiang, Xiaomei Deng, Sihan Li, Xiaojun Zeng*, Chunxiao Wu*, Chao Yang*


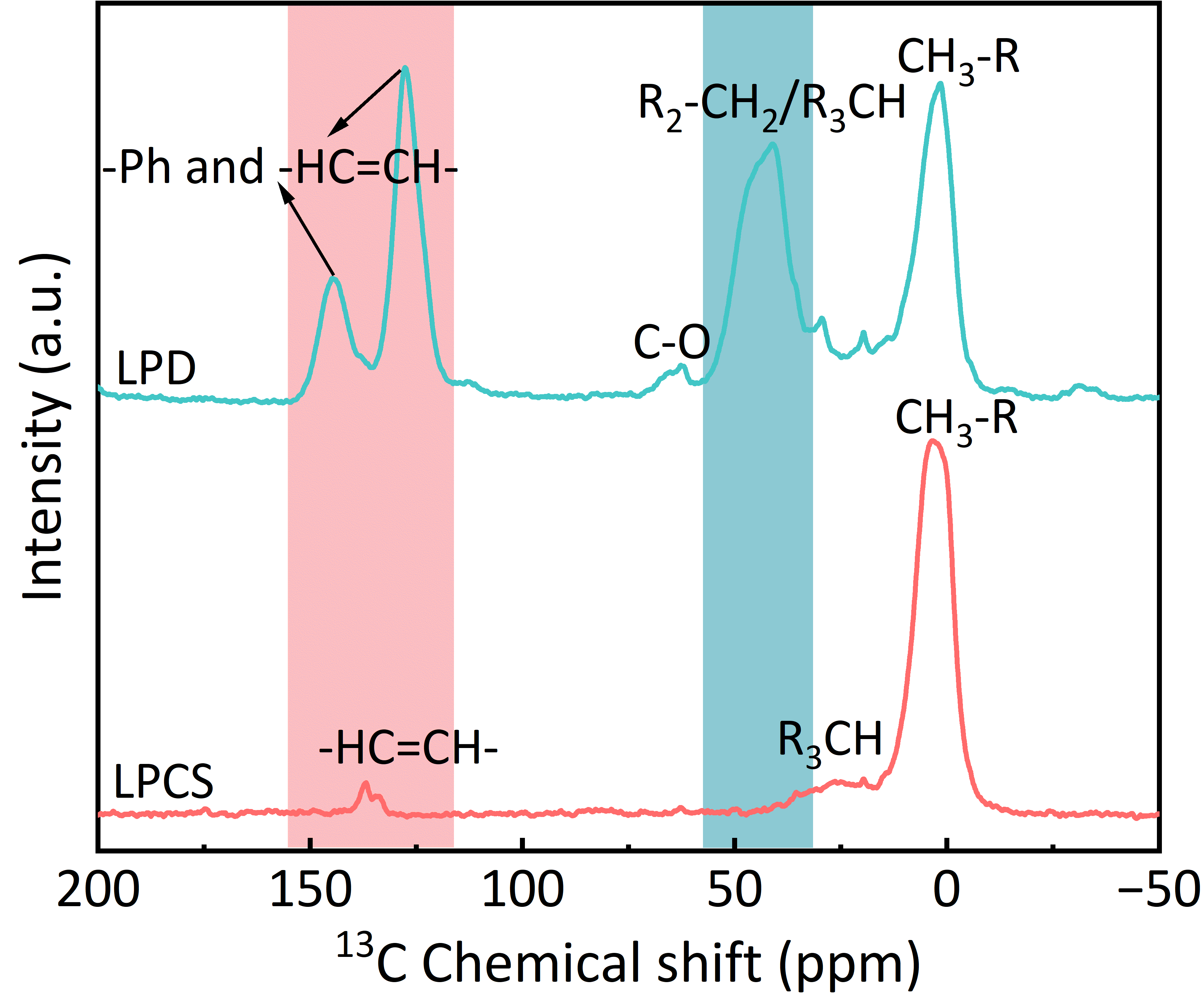


**Figure S1.**  Nuclear magnetic resonance spectra of ^13^C-NMR of LPD and LPCS.


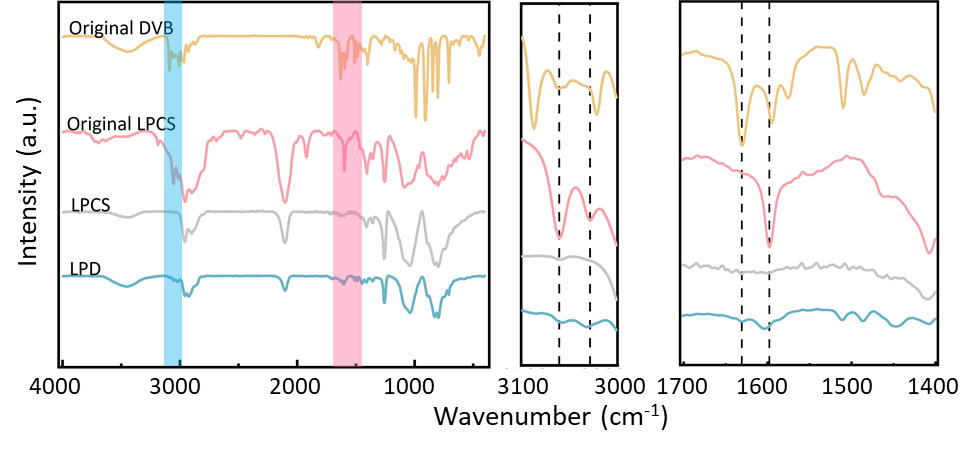


**Figure S2.** Fourier transform infrared spectroscopic analysis of DVB, LPCS, SPCS, and LPD.

A series of peaks serve as characteristics of LPCS, as illustrated below: 3055, 3015 cm^-1^(ν, C-H stretching in -CH=CH-), 2952 cm^−1^ (νas, C–H asymmetrical stretching in –CH_3_), 2898 cm^−1^ (νs, C–H symmetrical stretching in –CH_3_), 2106 cm^−1^ (ν, Si-H stretching), 1597 cm^−1^ (ν, C=C stretching), 1406, 1255 cm^−1^ (δ, Si–C deformation in –Si–CH_3_), and so on. Concurrently, DVB is characterized by 3088, 3007cm^-1^(ν, C-H stretching in -CH=CH- and phenyl), 1630 cm^−1^ (ν, C=C stretching in -CH=CH-), 1595, 1575, 1510 and 1485 cm^-1^(ν, C=C stretching in phenyl).

To investigate the structure of LPD, partially enlarged views of FTIR infrared spectroscopy ranging from 3100-2980 cm^-1^ and 1700-1400 cm^-1^ are portrayed in Figure S2-3. Figure S2-3 shows that LPD retains the characteristic peaks of C-H and C=C from LPCS, albeit with diminished intensities. Notably, characteristic peaks corresponding to C-H in DVB vanish, while peaks attributed to phenyl retain, implying that unsaturated bonds in LPCS and DVB react during the process. Potential reactions encompass hydrosilylation and vinyl polymerization reactions.


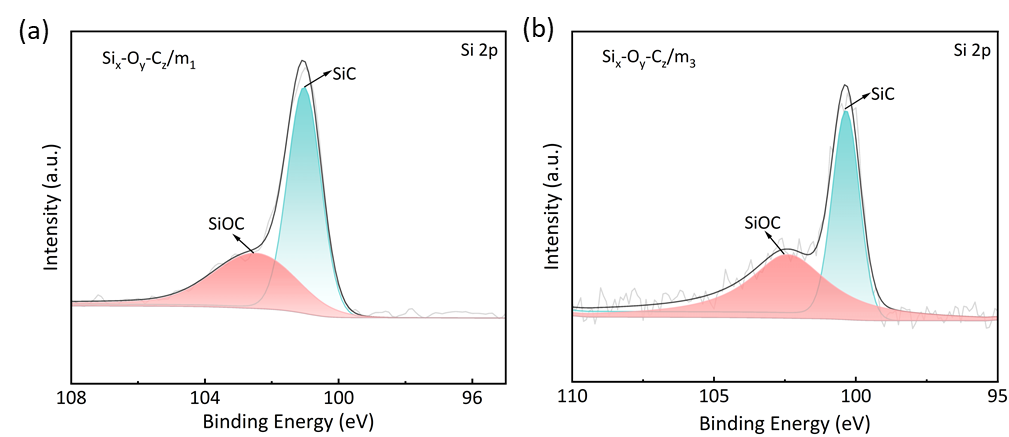


**Figure S3.** Si 2p XPS spectra of a) Si_x_-O_y_-C_z_/m_1_ and b) Si_x_-O_y_-C_z_/m_3_.


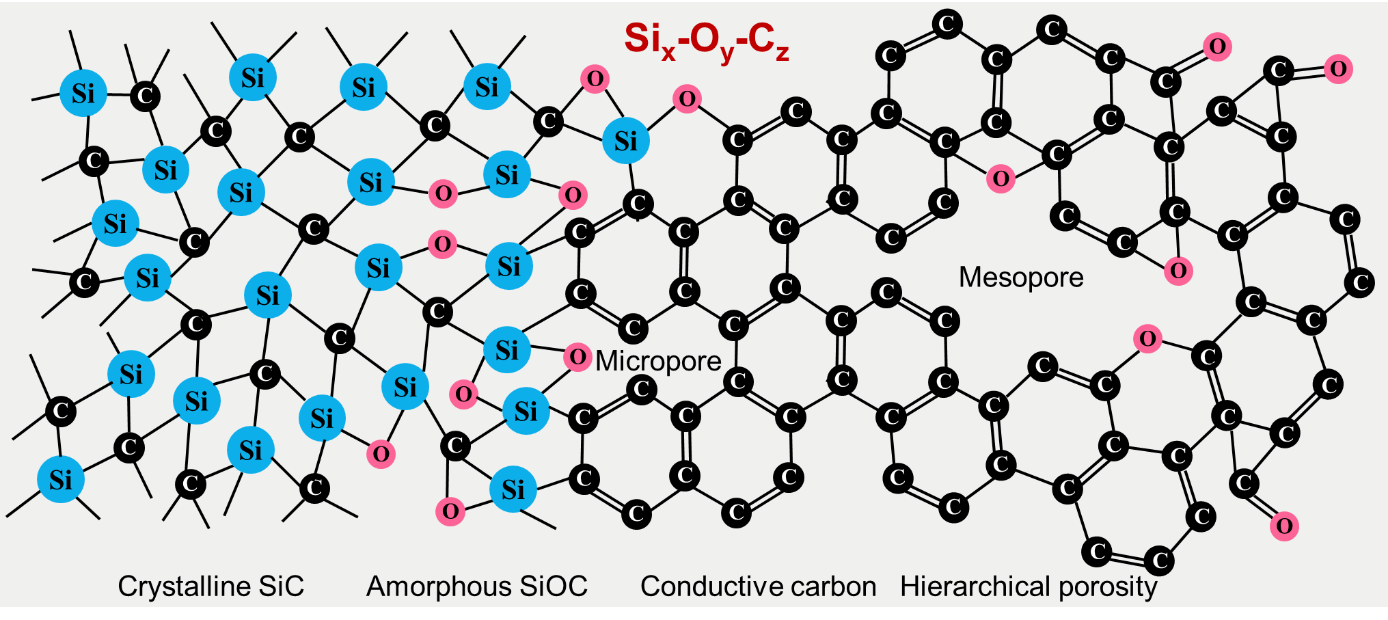


**Figure S4.** Chemical bonding structure of Si_x_-O_y_-C_z_.


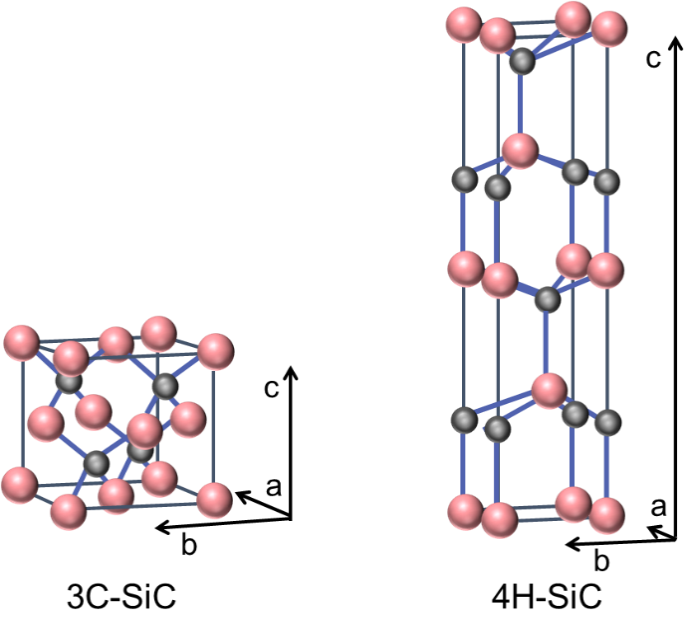


**Figure S5.** Representative atomic arrangements of 3C-SiC (cubic) and 4H-SiC (hexagonal) structures. Point defect Vc (vacancy) is also illustrated as a missing atom in the lattice.


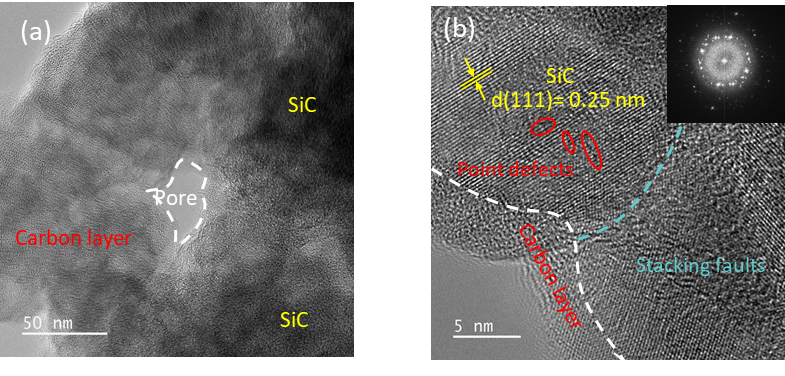


**Figure S6.** a) TEM image of Si_x_-O_y_-C_z_/m_1_ showing a representative hierarchical porous structure composed of SiC domains and surrounding carbon layers. A pore is clearly visible at the center, highlighting the etching-induced porosity. b) High-resolution TEM (HRTEM) image of Si_x_-O_y_-C_z_/m₁, illustrating the crystalline SiC lattice with an interplanar spacing of 0.25 nm corresponding to the (111) planes. Point defects and stacking faults are observed within the SiC domains, along with a distinct interface between SiC and the carbon layer. Inset: Selected area electron diffraction (SAED) pattern confirming the crystalline nature of SiC.


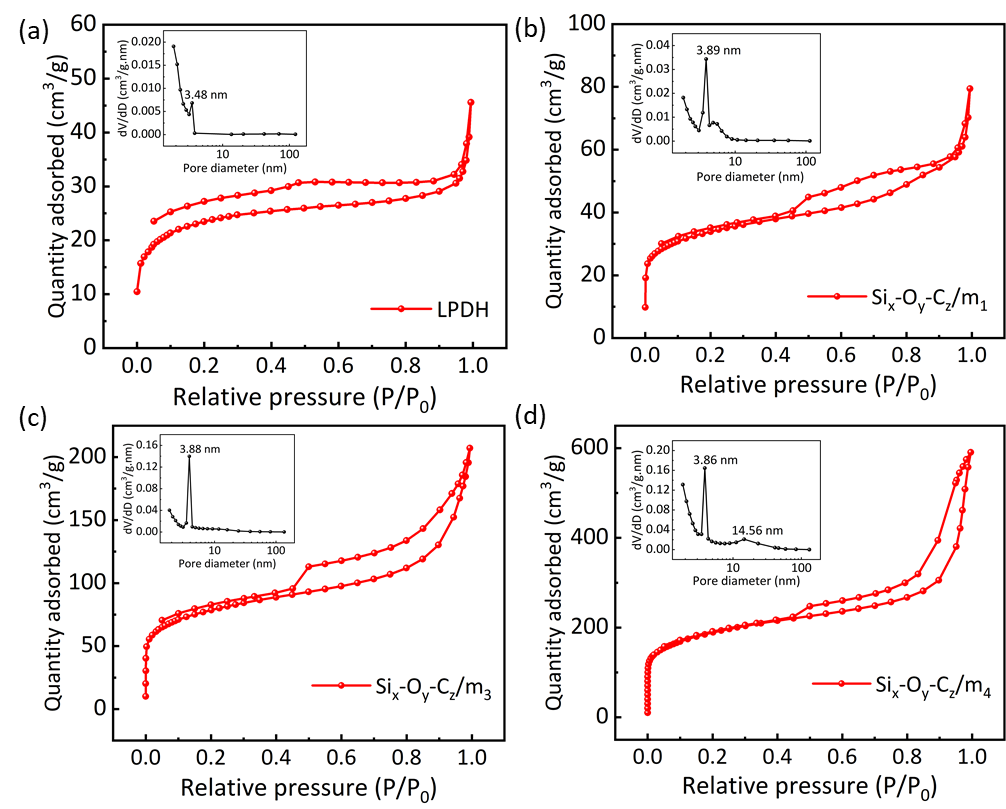


**Figure S7.** Nitrogen physisorption isotherms and pore size distribution of a) LPDH, b) Si_x_-O_y_-C_z_/m_1_, c) Si_x_-O_y_-C_z_/m_3_, and d) Si_x_-O_y_-C_z_/m_4_.


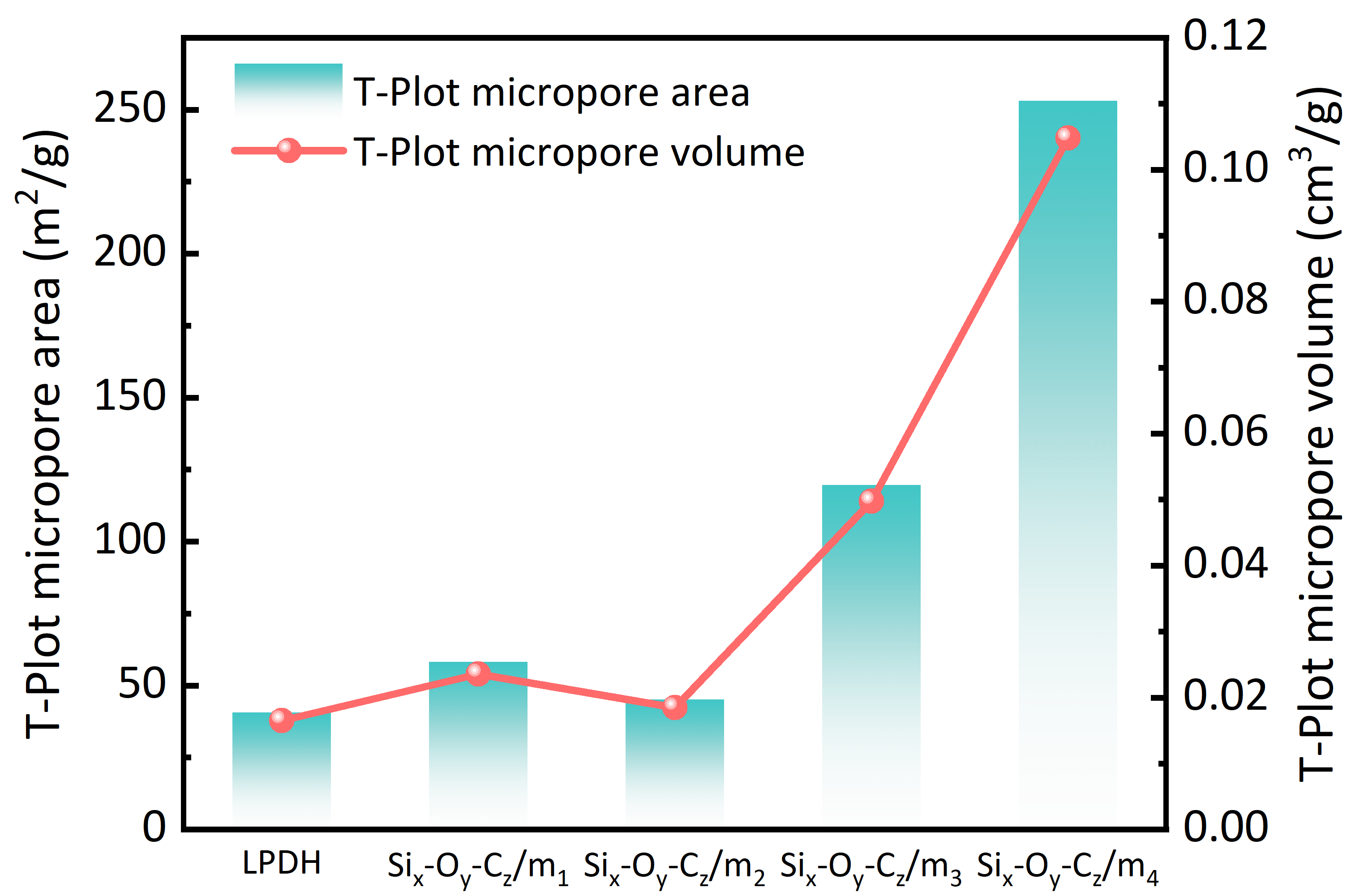


**Figure S8.** Quantitative analysis of microporous structures in Si_x_-O_y_-C_z_ samples using the t-plot method. Both micropore area (left axis) and micropore volume (right axis) show a consistent increasing trend with porosity, confirming the abundant micropore formation.


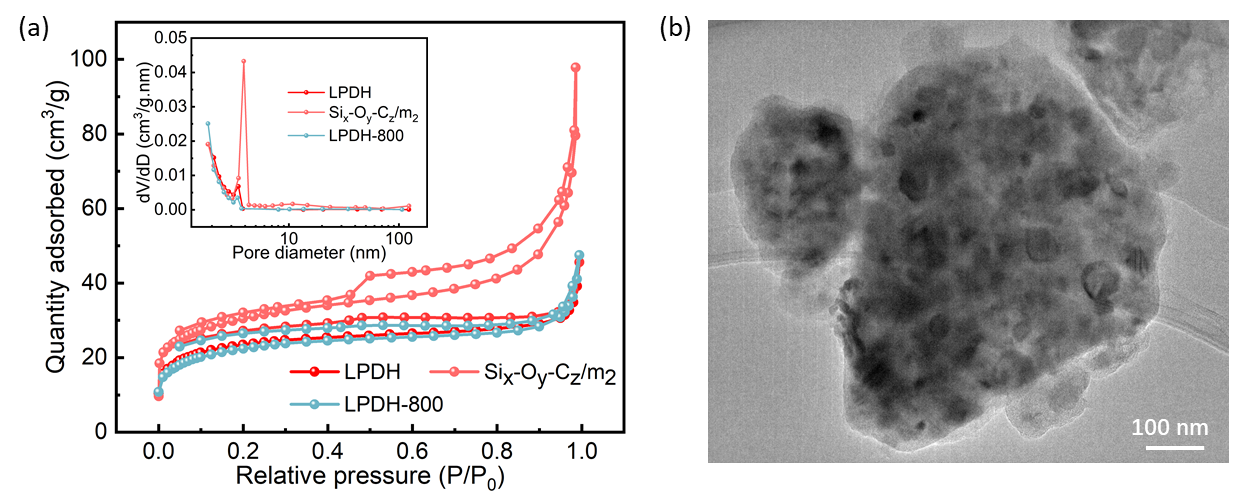


**Figure S9.** BET and TEM results of LPDH calcined at the same temperature (800 ℃) as Si_x_-O_y_-C_z_.


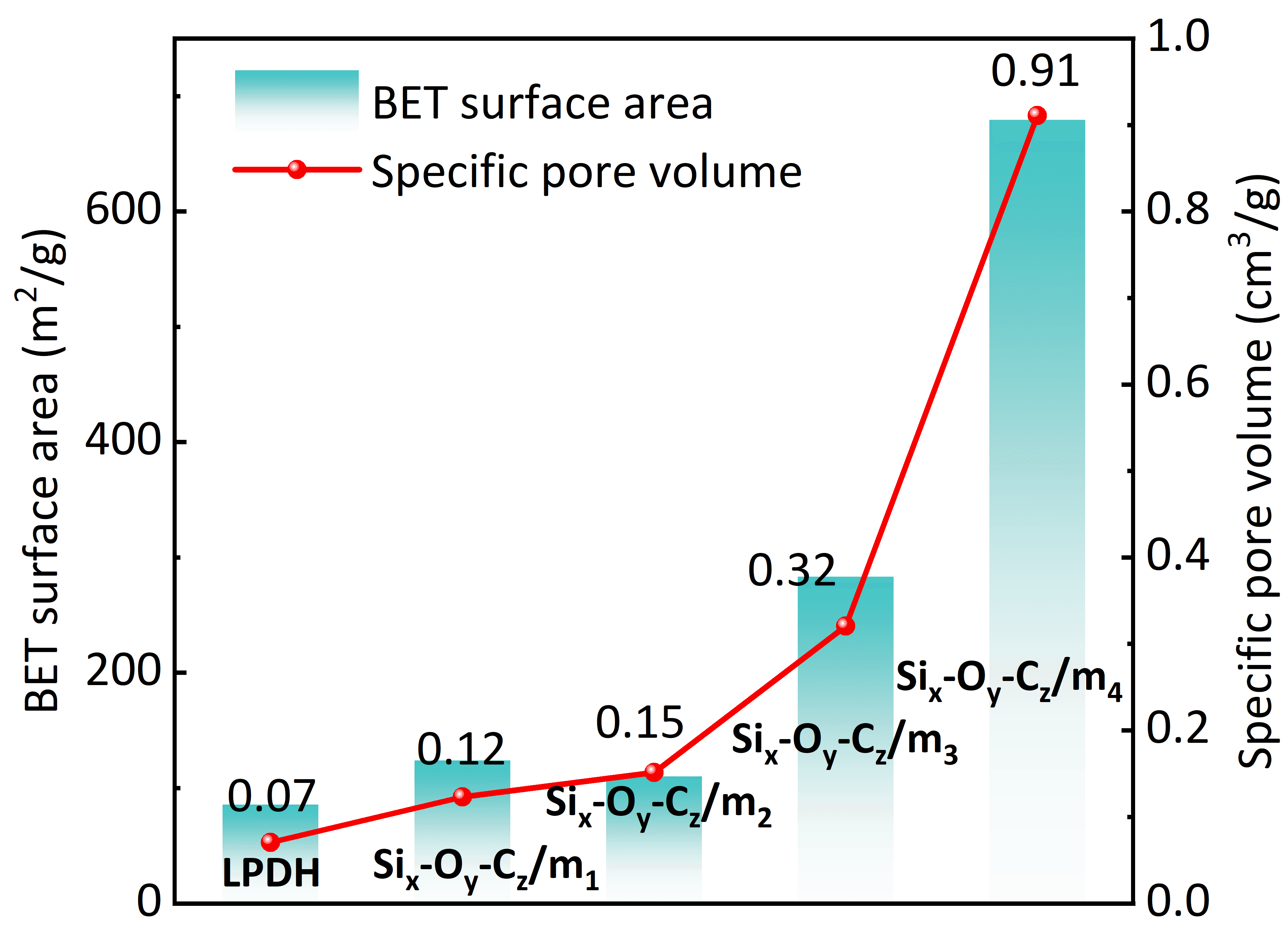


**Figure S10.** BET specific surface area and specific pore volume of LPDH, Si_x_-O_y_-C_z_/m_1_, Si_x_-O_y_-C_z_/m_2_, Si_x_-O_y_-C_z_/m_3_, and Si_x_-O_y_-C_z_/m_4_.


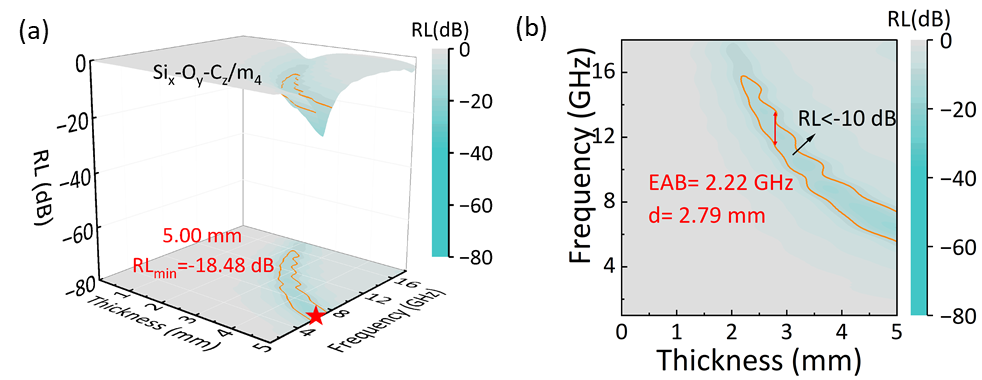


**Figure S11.** a) 3D and b) 2D patterns of Si_x_-O_y_-C_z_/m_4_.


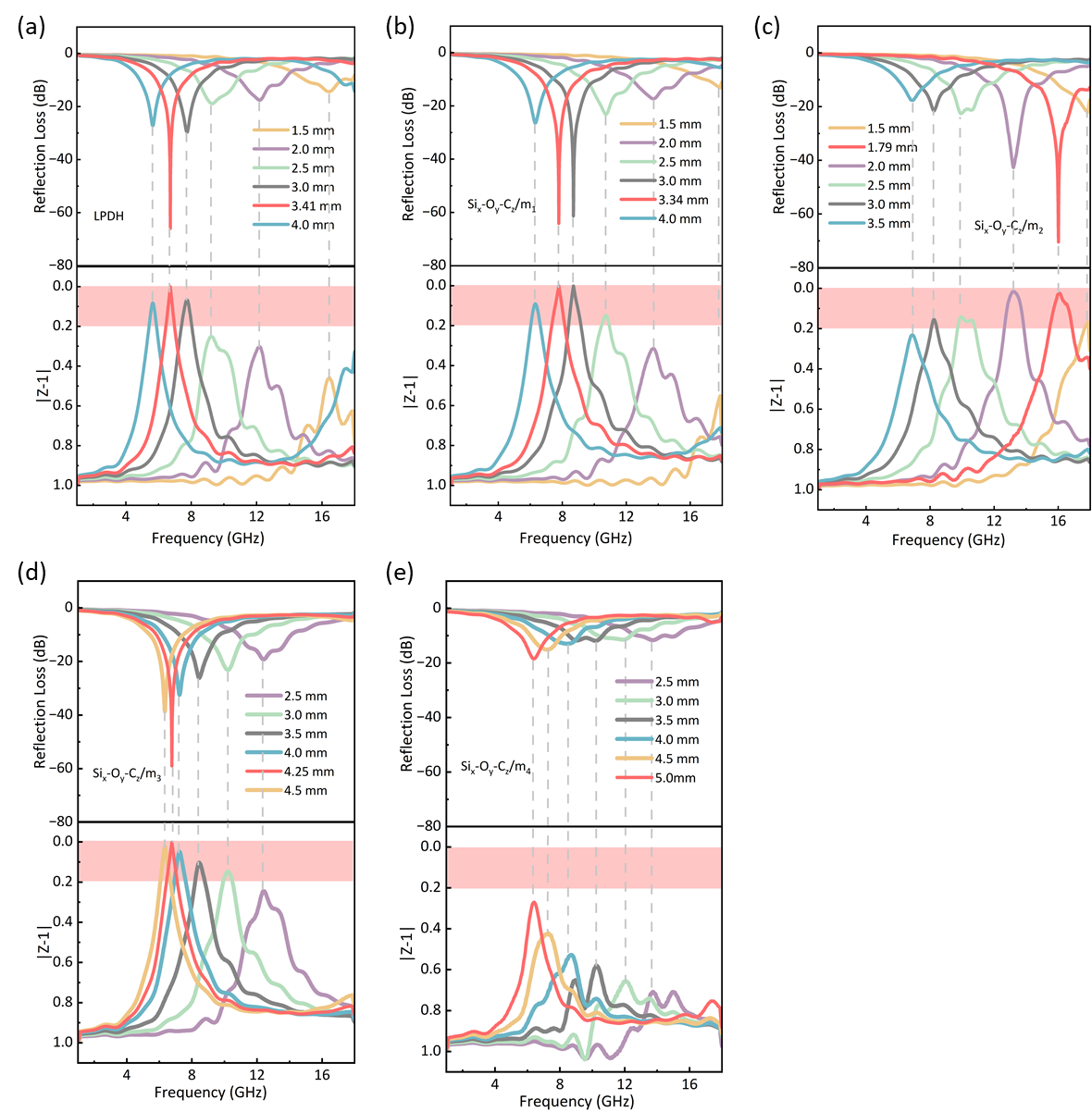


**Figure S12.** Reflection loss and impedance matching |Z-1| of all samples.

**
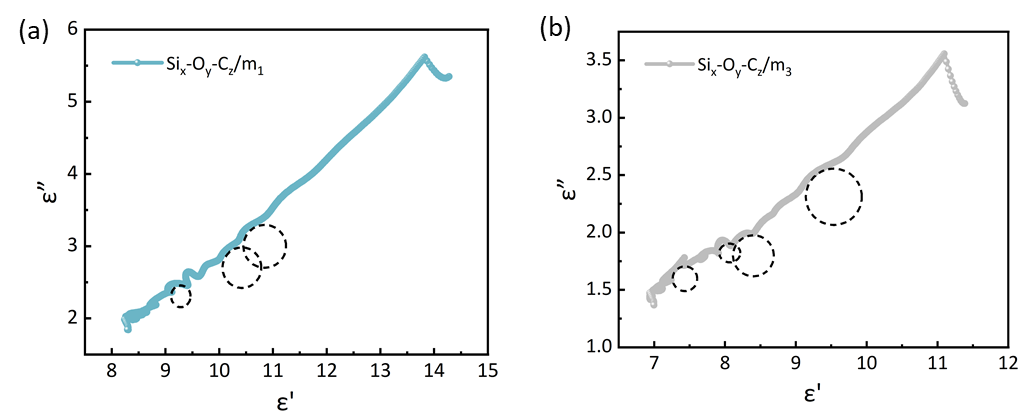
**

**Figure S13.** Cole-Cole plots of a) Si_x_-O_y_-C_z_/m_1_ and b) Si_x_-O_y_-C_z_/m_3_.


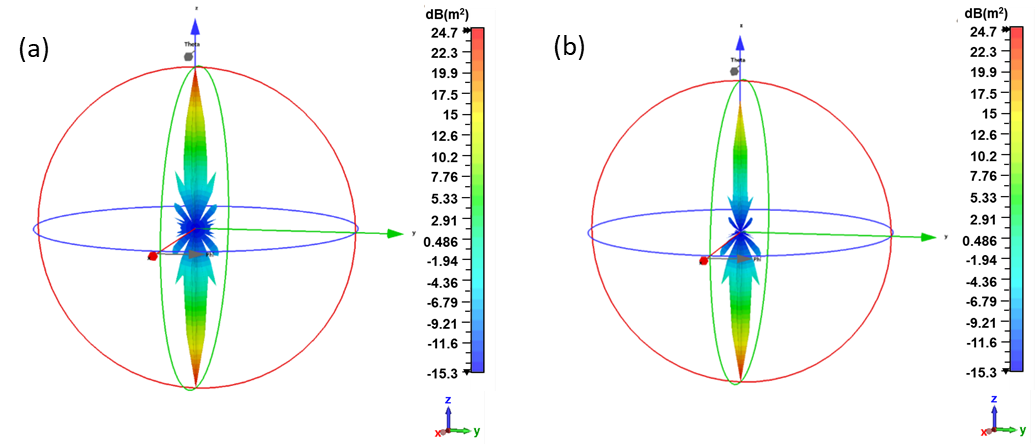


**Figure S14.** 3D radar wave scattering signals of a) PEC and b) Si_x_-O_y_-C_z_/m_4_.


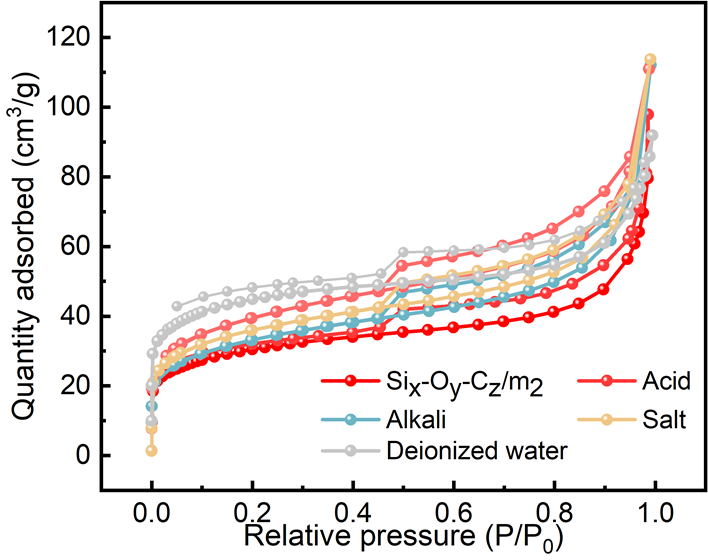


**Figure S15.** Nitrogen adsorption–desorption isotherms and pore size distributions of Si_x_-O_y_-C_z_/m_2_ immersed in acid (pH = 1), alkali (pH = 13), salt (5 wt.% NaCl), and deionized water for 7 days.


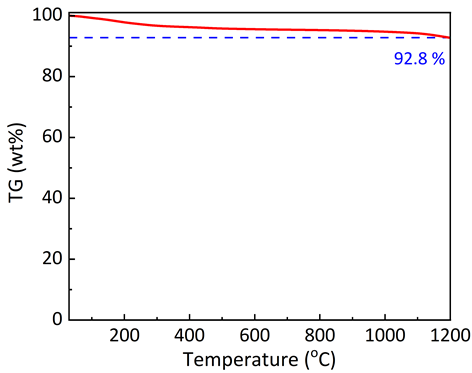


**Figure S16.** Thermogravimetric analysis (TGA) curve of Si_x_-O_y_-C_z_/m_2_ measured under Ar atmosphere up to 1200 °C.

**Table S1.** EMW absorption performance of Si_x_-O_y_-C_z_ and recently advanced ceramic-based absorbers

| No. | Sample | RL_min_(dB) | d(mm) | EAB(GHz) | RL_min_/d(dB/mm) | Ref. |
| --- | --- | --- | --- | --- | --- | --- |
| S1 | Fe/SiOC | -20.50 | 2.80 | 3.60 | -7.32 | ^[1]^ |
| S2 | Fe_x_Si_y_/SiC@SiOC | -38.00 | 4.80 | 2.60 | -7.92 | ^[2]^ |
| S3 | Si_3_N_4_/ERG | -26.70 | 3.25 | 4.20 | -8.22 | ^[3]^ |
| S4 | SiO_2_/SiC–Y_2_Si_2_O_7_ | -28.00 | 3.15 | 4.20 | -8.89 | ^[4]^ |
| S5 | Fe_3_Si@C/SiC/Fe_3_O_4_/SiO_2_ | -44.70 | 4.50 | - | -9.93 | ^[5]^ |
| S6 | LAS/N-GF | -47.98 | 4.50 | 8.34 | -10.66 | ^[6]^ |
| S7 | Nd_2_O_3_–SiOC | -45.12 | 4.15 | 4.20 | -10.87 | ^[7]^ |
| S8 | CNTs-SiCN | -21.80 | 2.00 | 3.70 | -10.90 | ^[8]^ |
| S9 | CNTs/Sc_2_Si_2_O_7_ | -33.50 | 2.85 | 4.20 | -11.75 | ^[9]^ |
| S10 | SiCnp@SiCf | -57.04 | 4.80 | 7.50 | -11.88 | ^[10]^ |
| S11 | SiCN /MWCNTs | -21.70 | 1.70 | 4.70 | -12.76 | ^[11]^ |
| S12 | Porous SiOC | -39.13 | 3.00 | 4.64 | -13.04 | ^[12]^ |
| S13 | PDC aerogel | -42.00 | 3.00 | 6.60 | -14.00 | ^[13]^ |
| S14 | Si_3_N_4_@WS_2_ | -65.03 | 4.35 | 4.20 | -14.95 | ^[14]^ |
| S15 | SiC/SiO_2_ | -57.22 | 3.65 | 9.40 | -15.68 | ^[15]^ |
| S16 | SiC/Si_3_N_4_/C | -53.00 | 3.35 | 3.02 | -15.82 | ^[16]^ |
| S17 | SiC/HfC_x_N_1–x_/C | -47.00 | 2.97 | 3.10 | -15.82 | ^[17]^ |
| S18 | SiC-CNF | -53.30 | 3.18 | - | -16.76 | ^[18]^ |
| S19 | ZIF67/SiCN | -46.40 | 2.70 | 3.00 | -17.19 | ^[19]^ |
| S20 | SiOC aerogel | -73.80 | 4.28 | 6.72 | -17.24 | ^[20]^ |
| S21 | Pr_3_Si_2_C_2_/SiCw | -48.12 | 2.71 | 5.04 | -17.76 | ^[21]^ |
| S22 | Si_3_N_4_–SiC/SiO_2_ | -51.90 | 2.80 | 4.16 | -18.54 | ^[22]^ |
| S23 | Fe_3_Si/Fe/CNTs/SiOCN | -65.30 | 3.43 | 6.00 | -19.04 | ^[23]^ |
| S24 | PSO/FeCl_3_ | -58.37 | 2.95 | 4.20 | -19.79 | ^[24]^ |
| S25 | SiC/Fe_3_Si/CNTs | -41.20 | 2.00 | 4.00 | -20.60 | ^[25]^ |
| S26 | CCFA | -55.66 | 2.70 | 8.24 | -20.61 | ^[26]^ |
| S27 | 3D-printed Si–O–C | -56.11 | 2.70 | 3.76 | -20.78 | ^[27]^ |
| S28 | GNS/PyC@SiBCN | -67.00 | 3.20 | 4.20 | -20.94 | ^[28]^ |
| S29 | SiBCN | -56.90 | 2.60 | 3.45 | -21.88 | ^[29]^ |
| S30 | (ZrO_2_ -SiO_2_ )f /ZrB_2_ -SiOC | -62.73 | 2.85 | 4.20 | -22.01 | ^[30]^ |
| S31 | SiCNnw/C/Si_3_N_4_ | -61.24 | 2.77 | 6.30 | -22.11 | ^[31]^ |
| S32 | SiC@SiO_2_ | -56.60 | 2.55 | 10.20 | -22.20 | ^[32]^ |
| S33 | MoS_2_/Si_3_N_4_ | -68.66 | 3.06 | 4.20 | -22.44 | ^[33]^ |
| S34 | FeSiAl@Al_2_O_3_@SiO_2_ | -56.71 | 2.50 | 3.40 | -22.68 | ^[34]^ |
| S35 | SiBCNHf | -46.29 | 2.00 | 7.33 | -23.15 | ^[35]^ |
| S36 | SiOC/ZrB_2_ | -29.30 | 1.26 | 13.50 | -23.25 | ^[36]^ |
| S37 | SiBCN/Al_2_O_3_ | -48.60 | 2.00 | 5.80 | -24.30 | ^[37]^ |
| S38 | SiBCNa/mullite/SiCnw | -40.30 | 1.60 | 3.40 | -25.19 | ^[38]^ |
| S39 | LAS/CNT | -50.49 | 2.00 | 5.40 | -25.25 | ^[39]^ |
| S40 | SiCN | -59.59 | 2.30 | 4.20 | -25.91 | ^[40]^ |
| S41 | SiBCN | -54.24 | 2.05 | 3.15 | -26.46 | ^[41]^ |
| S42 | Co/CNT@SiC | -64.16 | 2.28 | 8.02 | -28.14 | ^[42]^ |
| S43 | mullite anti-gyroid/SiC gyroid | -54.00 | 1.90 | 3.20 | -28.42 | ^[43]^ |
| S44 | SiBCN-rGO | -62.71 | 2.17 | 4.32 | -28.90 | ^[44]^ |
| S45 | CNT/CoSi/SiOC | -66.40 | 2.27 | 6.16 | -29.25 | ^[45]^ |
| S46 | SiBON/rGO | -50.43 | 1.70 | 4.80 | -29.66 | ^[46]^ |
| S47 | DLP-SiBCN | -70.60 | 2.31 | 3.09 | -30.56 | ^[47]^ |
| S48 | MoS_2_/SiCnw/Si_3_N_4_ | -70.48 | 2.10 | 3.50 | -33.56 | ^[48]^ |
| S49 | PCN/SiO_2_@MXene/Fe_3_C | -71.14 | 2.03 | 3.85 | -35.04 | ^[49]^ |
| S50 | SiOC@C | -62.76 | 1.63 | 4.79 | -38.50 | ^[50]^ |
| - | Si_x_-O_y_-C_z_ | -70.44 | 1.79 | 4.32 | -39.35 | This work |

**Reference:**

[1] Y. Ma, F. Yang, S. Kou, et al., Enhanced microwave absorption properties of Fe-doped SiOC ceramics by the magnetic-dielectric loss properties, 2021, Ceram. Int., 47, 24393, <https://doi.org/10.1016/j.ceramint.2021.05.153>

[2] C. Zhou, S. Li, Z. Yu, Polymer-derived Fe_x_Si_y_/SiC@SiOC ceramic nanocomposites with tunable microwave absorption behavior, 2021, Int J Appl Ceram Technol., 19, 813, <https://doi.org/10.1111/ijac.13850>

[3] F. Ye, Q. Song, Z. Zhang, et al., Direct Growth of Edge-Rich Graphene with Tunable Dielectric Properties in Porous Si_3_N_4_ Ceramic for Broadband High-Performance Microwave Absorption, 2018, Adv. Funct. Mater., 28, 1707205, <https://doi.org/10.1002/adfm.201707205>

[4] H. Wei, J. Liu, P. Feng, et al., Design of multilayer cauliflower-like structure SiO_2_/SiC–Y_2_Si_2_O_7_ composite ceramics as high-efficiency electromagnetic wave absorbers, 2022, Ceram. Int., 48, 33635, <https://doi.org/10.1016/j.ceramint.2022.07.309>

[5] C. Gu, C. Guo, X. Dong, et al., Core-shell structured iron-containing ceramic nanoparticles: Facile fabrication and excellent electromagnetic absorption properties, 2019, J. Am. Ceram. Soc., 102, 7098, <https://doi.org/10.1111/jace.16619>

[6] J. w. Qi, J. q. Zhang, Y. An, et al., Nitrogen-doped modified graphene aerogel enhancing interfacial bonding with lithium aluminium silicate ceramics for broadband microwave absorption, 2025, Carbon, 232, 119794, <https://doi.org/10.1016/j.carbon.2024.119794>

[7] C. Wang, H. Wang, L. Tang, J. Xue, Z. Wang, H. Wei, Vat photopolymerization 3D printed SiOC-based metamaterials with triply periodic minimal surface: Microwave absorption and load-bearing properties, 2025, Addit. Manuf., 104, 104776, <https://doi.org/10.1016/j.addma.2025.104776>

[8] X. Liu, Z. Yu, L. Chen, et al., Role of single-source-precursor structure on microstructure and electromagnetic properties of CNTs-SiCN nanocomposites, 2017, J. Am. Ceram. Soc., 100, 4649, <https://doi.org/10.1111/jace.15000>

[9] H. Wei, X. Yin, X. Li, et al., Controllable synthesis of defective carbon nanotubes/Sc_2_Si_2_O7 ceramic with adjustable dielectric properties for broadband high-performance microwave absorption, 2019, Carbon, 147, 276, <https://doi.org/10.1016/j.carbon.2019.03.008>

[10] Y. Hou, B. Chen, H. Zhang, et al., Co-electrospun SiC nanoparticle-decorated SiC fiber hybrid materials towards highly effective and broadband electromagnetic attenuation performance, 2025, Ceram. Int., S0272884225012878, Advance online publication, <https://doi.org/10.1016/j.ceramint.2025.03.170>

[11] S. Wang, H. Gong, M. Z. Ashfaq, D. Qi, X. Yue, Introducing MWCNTs conductive network in polymer-derived SiCN ceramics for broadband electromagnetic wave absorption, 2022, Ceram. Int., 48, 23989, <https://doi.org/10.1016/j.ceramint.2022.05.075>

[12] C. Chen, S. Zeng, X. Han, et al., 3D carbon network supported porous SiOC ceramics with enhanced microwave absorption properties, 2020, J Mater Sci Technol., 54, 223, <https://doi.org/10.1016/j.jmst.2020.03.018>

[13] W. Zhao, G. Shao, M. Jiang, et al., Ultralight polymer-derived ceramic aerogels with wide bandwidth and effective electromagnetic absorption properties, 2017, J. Eur. Ceram. Soc., 37, 3973, <https://doi.org/10.1016/j.jeurceramsoc.2017.04.068>

[14] J. Bai, S. Huang, X. Yao, X. Liu, Z. Huang, Core-shell Si_3_N_4_@WS_2_ porous ceramics with improved electromagnetic wave absorption performance, 2024, J. Mater. Chem. C., 12, 5776, <https://doi.org/10.1039/d3tc04672a>

[15] Z. Wang, J. Liu, H. Hao, et al., Microwave absorption enhancement by SiC nanowire aerogels through heat treatment-based oxidation modulation, 2024, Carbon, 217, 118622, <https://doi.org/10.1016/j.carbon.2023.118622>

[16] Q. Li, X. Yin, W. Duan, B. Hao, L. Kong, X. Liu, Dielectric and microwave absorption properties of polymer derived SiCN ceramics annealed in N_2_ atmosphere, 2014, J. Eur. Ceram. Soc., 34, 589, <https://doi.org/10.1016/j.jeurceramsoc.2013.08.042>

[17] Q. Wen, Y. Feng, Z. Yu, et al., Microwave Absorption of SiC/HfC_x_N_1–x_/C Ceramic Nanocomposites with HfC_x_N_1–x_-Carbon Core-Shell Particles, 2016, J. Am. Ceram. Soc., 99, 2655, <https://doi.org/10.1111/jace.14256>

[18] B. Du, D. Zhang, J. Qian, et al., Multifunctional carbon nanofiber-SiC nanowire aerogel films with superior microwave absorbing performance, 2021, Adv. Compos. Hybrid Mater., 4, 1281, <https://doi.org/10.1007/s42114-021-00286-1>

[19] T.-B. Geng, G.-Y. Yu, G.-F. Shao, X.-G. Huang, Enhanced electromagnetic wave absorption properties of ZIF-67 modified polymer-derived SiCN ceramics by in situ construction of multiple heterointerfaces, 2023, Rare Met., 42, 1635, <https://doi.org/10.1007/s12598-023-02270-8>

[20] A. Yan, G. Li, Z. Su, et al., High-attenuation and broadband microwave absorption of robust and thermally stable SiOC ceramic aerogels derived from interpenetrating silicone double network structure, 2025, Chem. Eng. J., 514, 163274, <https://doi.org/10.1016/j.cej.2025.163274>

[21] H. Zhu, G. Qin, W. Zhou, Y. Li, X. Zhou, Constructing flake-like ternary rare earth Pr_3_Si_2_C_2_ ceramic on SiC whiskers to enhance electromagnetic wave absorption properties, 2024, Ceram. Int., 50, 134, <https://doi.org/10.1016/j.ceramint.2023.10.050>

[22] M. Li, X. Yin, G. Zheng, et al., High-temperature dielectric and microwave absorption properties of Si_3_N_4_-SiC/SiO_2_ composite ceramics, 2014, J. Mater. Sci., 50, 1478, <https://doi.org/10.1007/s10853-014-8709-y>

[23] Z. Yu, Q. Zhu, F. Li, T. Chen, H. Du, Single-source-precursor derived multicomponent CNTs/Fe_3_Si/Fe/SiOCN ceramic nanocomposites: microstructural evolution and excellent electromagnetic wave absorbing properties, 2022, J. Mater. Chem. C., 10, 6252, <https://doi.org/10.1039/d1tc05916e>

[24] D. Ding, J. Wang, G. Xiao, et al., Enhanced electromagnetic wave absorbing properties of Si-O-C ceramics with in-situ formed 1D nanostructures, 2019, Int J Appl Ceram Technol., 17, 734, <https://doi.org/10.1111/ijac.13338>

[25] Y. Hou, B. Xiao, Z. Sun, et al., High temperature anti-oxidative and tunable wave absorbing SiC/Fe_3_Si/CNTs composite ceramic derived from a novel polysilyacetylene, 2019, Ceram. Int., 45, 16369, <https://doi.org/10.1016/j.ceramint.2019.05.165>

[26] W. Huang, Y. Yang, H. Gu, W. Yu, G. Shao, A core-shell carbon-ceramic fibrous aerogel derived from aramid-polysilsesquioxane for broadband electromagnetic wave absorption, 2025, J. Mater. Chem. C., 13, 10658, <https://doi.org/10.1039/d5tc01397f>

[27] R. Zhou, Y. Wang, Z. Liu, Y. Pang, J. Chen, J. Kong, Digital Light Processing 3D-Printed Ceramic Metamaterials for Electromagnetic Wave Absorption, 2022, Nano-Micro Lett., 14, 122, <https://doi.org/10.1007/s40820-022-00865-x>

[28] L. Du, Y. Li, Q. Zhou, et al., Facile preparation of GNS/PyC@SiBCN aerogels with heterogeneous interfaces for broadband and tunable electromagnetic wave absorption, 2025, Mater. Today Nano, 29, 100571, <https://doi.org/10.1016/j.mtnano.2025.100571>

[29] Q. Chen, D. Li, X. Liao, et al., Polymer-Derived Lightweight SiBCN Ceramic Nanofibers with High Microwave Absorption Performance, 2021, ACS Appl. Mater., 13, 34889, <https://doi.org/10.1021/acsami.1c07912>

[30] Y. Deng, B. Ren, Y. Jia, Q. Wang, H. Li, Layered composites made of polymer derived SiOC/ZrB_2_ reinforced by ZrO_2_/SiO_2_ fibers with simultaneous microwave absorption and thermal insulation, 2024, J Mater Sci Technol., 196, 50, <https://doi.org/10.1016/j.jmst.2023.12.053>

[31] J. Bai, Z. Xie, P. Zhang, et al., Porous SiCNnw/C/Si_3_N_4_ ceramics with controlled component and structure for electromagnetic wave absorption, 2025, Compos. Part B Eng., 294, 112164, <https://doi.org/10.1016/j.compositesb.2025.112164>

[32] Z. Wang, Y. Hou, H. Hao, Y. Shuai, Z. Wang, Scalable preparation of SiC@SiO_2_ nanocable aerogels for broadband microwave absorption using low-cost carbon source, 2023, Carbon, 211, 118092, <https://doi.org/10.1016/j.carbon.2023.118092>

[33] J. Bai, S. Huang, X. Yao, X. Liu, Z. Huang, Surface engineering of nanoflower-like MoS_2_ decorated porous Si_3_N_4_ ceramics for electromagnetic wave absorption, 2023, J. Mater. Chem. A., 11, 6274, <https://doi.org/10.1039/d3ta00122a>

[34] Y. Guo, X. Jian, L. Zhang, et al., Plasma-induced FeSiAl@Al_2_O_3_@SiO_2_ core-shell structure for exceptional microwave absorption and anti-oxidation at high temperature, 2020, Chem. Eng. J., 384, 123371, <https://doi.org/10.1016/j.cej.2019.123371>

[35] Y. Song, Z. Liu, X. Zhang, et al., Single source precursor derived SiBCNHf ceramic with enhanced high-temperature microwave absorption and antioxidation, 2022, J Mater Sci Technol., 126, 215, <https://doi.org/10.1016/j.jmst.2022.03.015>

[36] Y. Jia, M. A. R. Chowdhury, D. Zhang, C. Xu, Wide-Band Tunable Microwave-Absorbing Ceramic Composites Made of Polymer-Derived SiOC Ceramic and in Situ Partially Surface-Oxidized Ultra-High-Temperature Ceramics, 2019, ACS Appl. Mater., 11, 45862, <https://doi.org/10.1021/acsami.9b16475>

[37] J. Jiang, L. Yan, J. Li, et al., Lightweight, thermally insulating SiBCN/Al_2_O_3_ ceramic aerogel with enhanced high-temperature resistance and electromagnetic wave absorption performance, 2024, Chem. Eng. J., 501, 157656, <https://doi.org/10.1016/j.cej.2024.157656>

[38] J. Jiang, L. Yan, J. Li, et al., Lightweight and resilient SiBCNa/mullite/SiCnw composite for thermal insulation and electromagnetic wave absorption, 2025, Ceram. Int., 51, 2315, <https://doi.org/10.1016/j.ceramint.2024.11.211>

[39] G. Ma, L. Xia, H. Yang, et al., Multifunctional lithium Aluminosilicate/CNT composite for gas filtration and electromagnetic wave absorption, 2021, Chem. Eng. J., 418, 129429, <https://doi.org/10.1016/j.cej.2021.129429>

[40] Y. Song, L. He, X. Zhang, et al., Highly Efficient Electromagnetic Wave Absorbing Metal-Free and Carbon-Rich Ceramics Derived from Hyperbranched Polycarbosilazanes, 2017, J. Phys. Chem. C., 121, 24774, <https://doi.org/10.1021/acs.jpcc.7b07646>

[41] Y. Wang, C. Luo, Y. Wu, et al., High temperature stable, amorphous SiBCN microwave absorption ceramics with tunable carbon structures derived from divinylbenzene crosslinked hyperbranched polyborosilazane, 2023, Carbon, 213, 118189, <https://doi.org/10.1016/j.carbon.2023.118189>

[42] W. Dong, M. Wei, S. U. Rehman, et al., Multifactor optimization of microwave absorption properties in Co/CNT@SiC composites via temperature-driven structural and compositional modulation, 2025, J. Mater. Chem. A., Advance Article, <https://doi.org/10.1039/d5ta01455g>

[43] C. Wang, X. Chen, Z. Wang, et al., A novel mullite anti-gyroid/SiC gyroid ceramic metastructure based on digital light processing 3D printing with enhanced electromagnetic wave absorption and mechanical properties, 2024, J. Adv. Ceram., 13, 1212, <https://doi.org/10.26599/jac.2024.9220930>

[44] Q. Chen, D. Li, Z. Yang, et al., SiBCN-reduced graphene oxide (rGO) ceramic composites derived from single-source-precursor with enhanced and tunable microwave absorption performance, 2021, Carbon, 179, 180, <https://doi.org/10.1016/j.carbon.2021.03.057>

[45] Z. Yu, T. Chen, H. Du, F. Li, Q. Zhu, Single-source-precursor derived SiOC ceramics with in-situ formed CNTs and core-shell structured CoSi@C nanoparticles towards excellent electromagnetic wave absorption properties, 2023, J. Adv. Ceram., 12, 1119, <https://doi.org/10.26599/jac.2023.9220743>

[46] H. Li, T. Zhang, J. Zhang, et al., Amorphism SiBON interface anchored rGO nanoplatelets composites with tunable electromagnetic properties for microwave absorption, 2023, Carbon, 214, 118343, <https://doi.org/10.1016/j.carbon.2023.118343>

[47] C. Liu, X. Guo, Y. Tong, et al., Vat photopolymerization 3D printing SiBCN ceramic metamaterials with strong electromagnetic wave absorption, 2024, Addit. Manuf., 87, 104239, <https://doi.org/10.1016/j.addma.2024.104239>

[48] J. Bai, S. Huang, X. Yao, X. Liu, Z. Huang, Construction of the SiC nanowires network structure decorated by MoS_2_ nanoflowers in porous Si_3_N_4_ ceramics for electromagnetic wave absorption, 2023, Chem. Eng. J., 469, 143809, <https://doi.org/10.1016/j.cej.2023.143809>

[49] J. Huang, X. Zeng, X. Jiang, et al., Assembly of SiO_2_@MXene spheres in PCN/Fe_3_C nanofibers with multifunctional electromagnetic wave absorption, 2025, Chem. Eng. J., 503, 158520, <https://doi.org/10.1016/j.cej.2024.158520>

[50] J. Qian, D. Ma, X. Zhou, et al., Synthesis of SiOC@C ceramic nanospheres with tunable electromagnetic wave absorption performance, 2024, J. Adv. Ceram., 13, 1394, <https://doi.org/10.26599/jac.2024.9220944>
